# Extracellular vesicles from pancreatic cancer and its tumour microenvironment promote increased Schwann cell migration

**DOI:** 10.1038/s41416-024-02915-0

**Published:** 2025-01-25

**Authors:** Fang Cheng Wong, Sebastian R. Merker, Lisa Bauer, Yi Han, Van Manh Hung Le, Carina Wenzel, Lukas Böthig, Max Heiduk, Pascal Drobisch, Venkatesh Sadananda Rao, Farzaneh Malekian, Ana Mansourkiaei, Christian Sperling, Heike Polster, Mathieu Pecqueux, Rouzanna Istvanffy, Linhan Ye, Bo Kong, Daniela E. Aust, Gustavo Baretton, Lena Seifert, Adrian M. Seifert, Jürgen Weitz, Ihsan Ekin Demir, Christoph Kahlert

**Affiliations:** 1 Department of Visceral, Thoracic and Vascular Surgery, University Hospital and Faculty of Medicine Carl Gustav Carus, Technische Universität Dresden, Dresden, Germany; 2 National Center for Tumor Diseases (NCT/UCC), Dresden, Germany; 3 German Cancer Research Center (DKFZ), Heidelberg, Germany; 4 Faculty of Medicine and University Hospital Carl Gustav Carus, Technische Universität Dresden, Dresden, Germany; 5 Helmholtz-Zentrum Dresden - Rossendorf (HZDR), Dresden, Germany; 6 Institute for Pathology, University Hospital Carl Gustav Carus Dresden, Technische Universität Dresden, Dresden, Germany; 7 Department of General, Visceral and Transplantation Surgery, University Hospital Heidelberg, Heidelberg, Germany; 8 German Cancer Consortium (DKTK), German Cancer Research Center (DKFZ), Heidelberg, Germany; 9 Department of Surgery, Klinikum rechts der Isar, Technical University of Munich, Munich, Germany; 10 German Cancer Consortium (DKTK), Partner Site Munich, Munich, Germany; 11 CRC 1321 Modelling and Targeting Pancreatic Cancer, Munich, Germany; 12 Tumour and Normal Tissue Bank of the University Cancer Center (UCC), University Hospital and Faculty of Medicine Carl Gustav Carus, Technische Universität Dresden, Dresden, Germany; 13 Else Kröner Clinician Scientist Professor for “Translational Tumor Immunological Research”, Dresden, Germany; 14 Department of General Surgery, HPB-Unit, School of Medicine, Acibadem Mehmet Ali Aydinlar University, Istanbul, Turkey; 15 Else Kröner Clinician Scientist Professor for “Translational Pancreatic Surgery”, Munich, Germany

**Keywords:** Pancreatic cancer, Cancer microenvironment, Metastasis

## Abstract

**Background:**

Pancreatic ductal adenocarcinoma (PDAC) exhibits a high frequency of neural invasion (NI). Schwann cells (SCs) have been shown to be reprogrammed to facilitate cancer cell migration and invasion into nerves. Since extracellular vesicles (EVs) affect the tumour microenvironment and promote metastasis, the present study analysed the involvement of EVs from pancreatic cancer cells and their microenvironment in altering SC phenotype as part of the early events in the process of NI.

**Methods:**

EVs were isolated from human/murine PDAC cells, pancreatic stellate cells (PSCs), human tissues and plasma to perform a novel 3D migration assay, qRT-PCR and western blot. Kaplan–Meier and Cox regression analyses were employed to evaluate the clinical potential of plasma EV-derived candidate from 165 PDAC patients.

**Results:**

The EVs from PDAC cells, PSCs derived from human tumour tissues, other cell types in the tumour microenvironment from tumour tissues and circulating plasma act as drivers of a pro-migratory phenotype of SCs by inducing dedifferentiation in SCs. Notably, p75NTR expression was upregulated in the plasma-derived EVs from patients with NI (Pn1) relative to those without NI (Pn0). High expression of plasma-derived EV p75NTR correlated with reduced overall survival and was identified as an independent prognostic factor.

**Conclusions:**

These findings suggest that EV-mediated SC migration underlies the interactions contributing to PDAC-associated NI with implications for improved outcome and therapeutic strategy.

## Introduction

Pancreatic ductal adenocarcinoma (PDAC) exhibits an exceptionally high frequency of neural invasion (NI), i.e., up to 100%, which has become one of the hallmarks of PDAC [1]. This special route of metastasis which involves penetration of cancer cells into, around or through the nerves has been associated with pain generation, an elevated risk of local recurrence and reduced patient survival [1–4]. Although the occurrence of NI is the most consistent in PDAC patients relative to other types of cancers [1], the mechanisms underlying NI have not been largely explored and there is currently no treatment strategy targeting NI prevention.

To understand the complex network of the tumour and its microenvironment in NI, several important findings have laid the foundation for gaining further insight into this clinical problem. Schwann cells (SCs) have been reported to play a vital role in promoting cancer invasion, which is beyond their classical role of providing trophic support for neurons in the peripheral nervous system (PNS) [5, 6]. SCs were found to be present in the human PanIN lesions and PDAC, and they were chemoattracted towards the cancer cells before the onset of cancer invasion resulting in the concept of “SC carcinotropism” [7], suggesting their involvement in the early stage of NI in PDAC and two-way interactions between cancer and SCs. In addition, SCs also interact with pancreatic cancer cells, intercalate between them and disperse them to promote NI [5]. Recently, reprogrammed SCs were demonstrated to be activated by cancer cells in a c-Jun dependent manner and provided an active pathway called tumour-activated Schwann cells tracks (TAST) for cancer cell migration and eventually invasion into neurons [8]. Since PDAC is notoriously difficult to treat because of the extremely dense desmoplastic reaction caused by myofibroblastic pancreatic stellate cells (PSCs), the role of PSCs in NI has also been described. For instance, hepatocyte growth factors (HGFs) released by PSCs promoted PDAC cell migration and invasion towards dorsal root ganglion (DRGs) by activating the HGF/c-Met pathway [9]. Taken together, it appears clear that there are active interactions in potentially multiple ways between SCs/PSCs and cancer cells driving NI in PDAC.

To decipher the interactions between different cell types, an emerging interest has focused on the key intercellular mediators, extracellular vesicles (EVs), which refer to nano-sized membrane-bound vesicles that naturally occur in the human body secreted by virtually every cell type. They carry biological cargoes (nucleic acids, proteins and lipids) orchestrating the key steps involved in initiating metastasis to eventual adaptation and propagation of tumour cells at the secondary sites in an organ-specific manner [10–12]. In the context of nerve microenvironment, a recent study shows that exosomes from human plasma and tissues of head and neck squamous cell carcinoma (HNSCC) induced neurite outgrowth of PC12, a rat pheochromocytoma cell line *via* exosomal EphrinB1, implying the potential of cancer-derived exosomes in promoting tumour innervation [13]. However, no study thus far elucidates the role of EVs in PDAC-associated NI.

This present study aimed to investigate the role of EVs from pancreatic cancer cells and their microenvironment in driving SC phenotype as part of an early event in the process of NI. Using a novel 3D migration assay incorporating EVs and human SCs (hSCs) in a single Matrigel drop, EVs derived from pancreatic cancer cells, Panc1 and tPSCs were demonstrated to increase the migratory ability of hSCs significantly. With the help of murine pancreatic cancer cell model [14], murine SCs (mSCs) were found to be dedifferentiated when confronted to neuroinvasive cancer cells compared to non-neuroinvasive cancer cells, as indicated by the upregulation of p75 neurotrophin receptor (p75NTR/NGFR) expression levels, which has been shown to play a vital role in mediating neuronal signalling and NI [7, 15, 16]. This was accompanied by the fact that p75NTR was enriched in the EVs of neuroinvasive cancer cells. At a translational aspect, the increased migration of hSCs was also observed after exposure to the tumour tissue-derived EVs from PDAC patients pathologically diagnosed with NI (Pn1) compared to those diagnosed without NI (Pn0). This was associated with an increased mRNA level of *NGFR*, indicating the relevance of neuroinvasive cancer-derived EVs in mediating SC migration. More importantly, high p75NTR expression in plasma-derived EVs was significantly associated with patients with Pn1, reduced overall survival (OS) and was demonstrated to be an independent unfavourable prognostic factor for PDAC prognosis. This study describes a previously unknown pro-migratory role of PDAC- and its tumour microenvironment-derived EVs in promoting SC migration that could foster the development of NI in PDAC patients.

## Materials and methods

### Experimental design and ethical approval

This was an analytical and experimental study which aimed to first investigate the role of EVs in altering the SC phenotype in the process of PDAC-associated NI. EVs from multiple sources such as human/murine pancreatic cancer cell lines, PSCs, human patient tissues and plasma were employed to elucidate the effect of EVs from not only the pancreatic cancer cells but also their microenvironment on SC migration. EVs were isolated by differential ultracentrifugation and well characterised by transmission electron microscopy (TEM), nanoparticle tracking analysis (NTA) and western blot. A novel 3D migration assay incorporating SCs and EVs was established to determine the effect of EVs from different sources in the same manner. The secondary objective was to identify a potential EV biomarker for diagnosing NI and predicting the OS of PDAC patients. Since plasma remains the most accessible source for liquid biopsy, plasma-derived EVs were isolated by size-exclusion chromatography (SEC) and protein candidate was detected by western blot. Kaplan–Meier curve and univariate and multivariate Cox regression analyses were employed to evaluate the performance of the candidate.

The whole study involved a total of 354 PDAC patients recruited at the Department of Visceral, Thoracic and Vascular Surgery, University Hospital Carl Gustav Carus (UHD), Technische Universität (TU) Dresden, Dresden, Germany after approval by the local ethics committee (EK76032013; EK499122017). Written informed consent from the patients was obtained pre-operatively with the disclosure of research purpose. EVs were isolated from plasma and tissues from a group of 53 PDAC patients for 3D migration assay using differential ultracentrifugation. Pancreatic cancer specimens and corresponding adjacent normal tissues were obtained from PDAC patients at UHD, Dresden, Germany with informed consent. The protocol was approved by the local ethics committee of the UHD (EK499122017). The characteristics of these 53 patients are presented in Table S1. In addition, primary cultures of PSCs isolated from pancreatic cancer tissues and corresponding adjacent normal tissues were approved by the same ethical approval. The details of the patients who provided the tissues for PSC isolation were presented in Supplementary Fig. 2E.

For analysis of the impact of NI status on OS and recurrence-free survival (RFS) of PDAC patients, a total of 304 patients were included and the clinical data of these patients were presented in Table S2. For identifying plasma-derived EV biomarkers, this investigation was designed as a retrospective exploratory study and complied with most of the Reporting Recommendations for Tumour Marker Prognostic Studies (REMARK) criteria [17]. Plasma samples from a total of 165 PDAC patients were received from the Department of Visceral, Thoracic and Vascular Surgery, UHD, Dresden, Germany after approval by the local ethics committee (EK76032013). The clinical information of PDAC patients for plasma-derived EV biomarker evaluation is presented in Table S3. The selection criteria for the analysis of NI status on OS and RFS as well as plasma-derived EV biomarkers were: (1) histologically verified PDAC; (2) identified NI status based on pathological reports; and (3) patients with a minimum age of 18 years. Clinical information included gender, age, tumour stage, grading, resection margin, neoadjuvant therapy, CA19-9 levels and follow-up time were collected. The clinical endpoint examined in this study was OS, which was defined as the time of surgery to the time of death or last follow-up. All the patients with OS shorter than 30 days were excluded to avoid cases of death due to surgical complications. The overview of patient selection for identifying plasma-derived EV biomarkers for diagnosis of NI and prognosis in PDAC patients was presented in Supplementary Fig. S1. All the experimental protocols used in this study were approved by the research lab at the Department of Visceral, Thoracic and Vascular Surgery, UHD, Dresden, Germany and all the methods were carried out in accordance with the relevant guidelines and regulations.

### 3D migration assay

hSCs/mSCs (1 × 10^5^ cells) mixed with respective pancreatic cell line/stellate cells/tissue-derived EVs (1 × 10^4^ particles per cell) in a ratio of 1:1 (25 μl Matrigel containing cells + 25 μl of SC basal medium containing EVs) were embedded in a single Matrigel drop (in a 24-well plate). The plate was incubated for 30 min at 37 °C for polymerisation, before adding 1 mL of SC basal medium supplemented with the same amount of EVs. The gel drops were monitored for a total of 4 days with half of the medium exchanged for fresh medium containing EVs after 2 days of initial seeding. Images were taken by digital phase contrast of Operetta CLS^TM^ high-content analysis system (PerkinElmer Inc., Waltham, Massachusetts, USA). Quantification was performed by using Harmony software provided by PerkinElmer Inc. to measure the area covered by cells (μm^2^). Briefly, the analysis sequence involved detecting cells (Find Nuclei), training to get the region of interest (Find Texture Regions; Texture A = positive cell area; Texture B = negative cell area) and expressing the region covered by cells in the area (μm^2^). The training step involved the selection of multiple positive areas defined as cells and negative areas defined as non-cells so as to obtain a region covered by cells.

For further details regarding other materials and methods used in this study, please refer to the Supplementary Information.

## Results

### EVs from pancreatic cancer cells and tumour-derived PSCs promote increased migration of hSCs

**Fig. 1:**
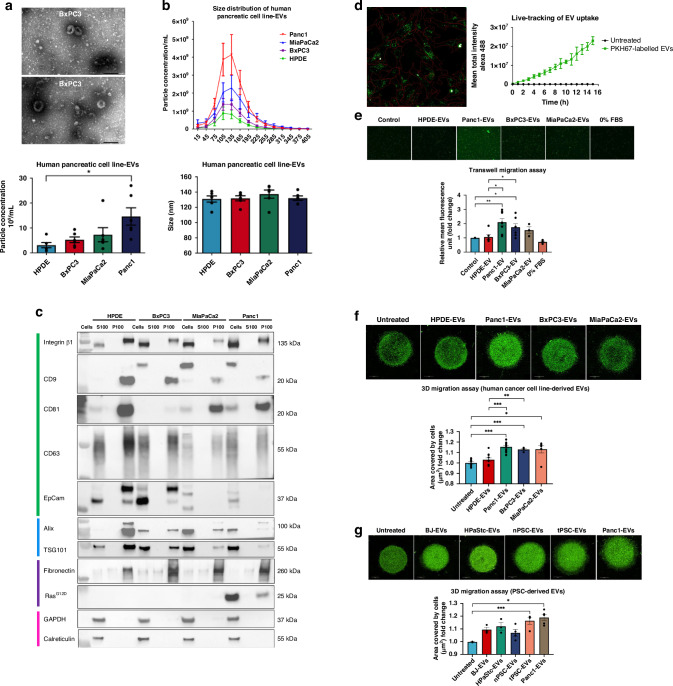
EVs from human pancreatic cancer and tPSCs promote increased migration of hSCs. **a** Representative negative-stain TEM images of BxPC3 pellet (P100) fractions. Scale bar indicates 200 nm. **b** NTA analysis of P100 fractions from HPDE, BxPC3, MiaPaCa2 and Panc1. Data represent the mean ± S.E.M. of six biological replicates. **c** Western blot analysis of EV and non-EV markers of proteins from whole cell lysates (Cells), EV-depleted supernatant (S100) and EV pellets from pancreatic cell lines (P100). Colour bars denote the EV marker categories from MISEV2018 guidelines: Category 1: Transmembrane proteins; Category 2: Cytosolic proteins; Category 4: Non-EV proteins; Category 5: Secreted proteins, e.g., cytokines, growth factors and ECM proteins. **d** Live-tracking analysis of PKH67-labelled Panc1-derived EV (green fluorescent spots) uptake by hSCs. Representative image of green fluorescent spots accumulated in the cells (red border) at time point 3. Line graphs illustrated the mean total intensity of Alexa Fluor 488 over 16 h. Measurements and analysis were performed by the Operetta CLS^TM^ high-content analysis system. **e** Transwell migration assay of hSCs exposed to EVs or control for 24 h. Untreated hSCs seeded onto the inserts placed in the wells containing EVs or control. Mean fluorescence unit was measured with ImageJ software. The data are mean ± S.E.M. from three independent experiments. **f**, **g** 3D migration assay of hSCs mixed with EVs from pancreatic cancer cell lines (**f**), BJ, HPaStc, nPSCs or tPSCs (**g**) embedded in a single Matrigel drop and monitored for 4 days. Images (pseudo-colour) were taken by digital phase contrast of the Operetta system. Quantification was performed by measuring the area covered by cells (µm^2^). The data are mean ± S.E.M. from three independent experiments. Statistical difference for all data was analysed by two-tailed unpaired Student’s *t*-test. *p < 0.05, **p < 0.01. HPDE Human pancreatic duct epithelial cells, BJ Fibroblasts derived from normal foreskin, HPaStc human pancreatic stellate cells, nPSC Pancreatic stellate cells derived from normal adjacent tissues, tPSC Pancreatic stellate cells derived from tumour tissue.

To test the hypothesis that EVs secreted by pancreatic cancer cells can trigger the migration of hSCs, EVs were isolated from BxPC3, MiaPaCa2, Panc1, and normal human pancreatic duct epithelial cells (HPDE) by differential ultracentrifugation. Characterisation of pancreatic cancer cells was performed by western blot (Supplementary Fig. 2A, B). These EVs were first characterised by TEM, NTA and western blot. TEM images reveal the vesicles isolated from BxPC3 were spherical and membrane-encapsulated with an artificial cup-shaped morphology, which is one of the typical features of EVs (Fig. 1a). Secondly, NTA shows that Panc1 cells secreted a significantly higher number of particles (14.62 × 10^9^ ± 3.47 particle/mL) compared to HPDE cells (3.10 × 10^9^ ± 1.03 particle/mL), given the same cell seeding density. The mean diameter distribution of particles from 4 cell lines was found to be around 130 nm (Fig. 1b), supporting the size definition of small EVs ranging from 30 nm to 150 nm. On top of that, EVs were subjected to protein composition characterisation based on Minimal information for studies of extracellular vesicles 2018 (MISEV2018) guidelines [18]. For transmembrane proteins, Integrin β1, CD9, CD81, CD63 and EpCAM were detected exclusively in the 100, 000 *x* g pellet (P100) fractions from HPDE, BxPC3, MiaPaCa2 and Panc1 cells, but not in the supernatant (S100) (Fig. 1c, Supplementary Fig. 3). Similar observation can be noticed for the category of cytosolic proteins related to MVEs, such as Alix and TSG101, as well as the category of secreted proteins, fibronectin. It is noteworthy that the RAS^G12D^ protein was clearly evident in the P100 fraction from the Panc1 cell line, which is known to carry *KRAS* point mutation (G > A) [19]. This indicates the fact that mutated proteins from cancer cell lines can be packaged into EVs. In addition, the absence of calreticulin, a marker of endoplasmic reticulum (ER), and GADPH, which has been proven to be present in the non-vesicular components, in the P100 fractions suggest the purity of the isolated EVs. Overall, the extensive characterisation results demonstrate that the isolated EVs correspond to the current notion of the EV identity.

After EV characterisation, the next step was to examine if hSCs could uptake EVs from pancreatic cancer cells. Hence, live-tracking analysis of Panc1-derived EVs labelled with PKH67 green fluorescent cell linker added onto hSCs was first conducted. It was observed that the green fluorescence signal increased throughout the treatment time of 15 h (Fig. 1d, Movie S1) and eventually accumulated at the perinuclear region of the cells (indicated as a red border) (Fig. 1d, left). Together with the phase contrast images and immunofluorescence (IF) staining of hSCs after the exposure to PKH67-labelled Panc1-EVs (Supplementary Fig. 4), these results confirm the time-dependent uptake of cancer cell-derived EVs by hSCs. Next, to investigate the functional effects of cancer cell-derived EVs on hSCs, a transwell migration assay was employed when untreated hSCs were placed in the insert of the transwell whereas pancreatic cell-derived EVs were added to the bottom of the wells. It was observed that there was a significant increase in the number of hSCs (illustrated by the increase in mean fluorescence unit) detected at the bottom of the inserts when Panc1- and BxPC3-derived EVs were present compared to control and HPDE-derived EVs, implying the chemoattraction displayed by the EVs (Fig. 1e). However, none of the human pancreatic cancer cell-derived EVs had an impact on the cell viability of hSCs across 3 different time points, i.e., 24 h, 48 h and 72 h (Supplementary Fig. 5). To allow for investigation in a more physiologically relevant condition, a novel 3D migration assay incorporated with EVs was first established in this study, in which pancreatic cell-derived EVs were mixed with hSCs in a single Matrigel drop. Consistent with the results from the 2D transwell migration assay, hSCs treated with Panc1- and BxPC3-derived EVs demonstrated the strongest migratory ability and were significantly different from the HPDE-derived EV-treated hSCs, as evidenced by larger area covered by cells (µm^2^) trained by the Harmony software associated with the Operetta CLS^TM^ high-content analysis system (Fig. 1f). Furthermore, only the hSCs exposed to tPSC-derived EVs increased the migratory ability significantly, but not the EVs derived from BJ, primary human pancreatic stellate cells (HPaStc) and PSCs derived from adjacent tissues (nPSCs) (Fig. 1g). The increased migration of hSCs exerted by tPSC-derived EVs was similar to that of the Panc1-derived EVs (Fig. 1g). These results show a specific cellular regulation in the migration of hSCs exerted by EVs from pancreatic cancer cell- and tumour-derived PSCs. Altogether, these data provide an indication that the migration of hSCs towards pancreatic cancer cells may be triggered by cancer cell- and PSC-derived EVs from pancreatic cancer and its tumour microenvironment.

**Fig. 2:**
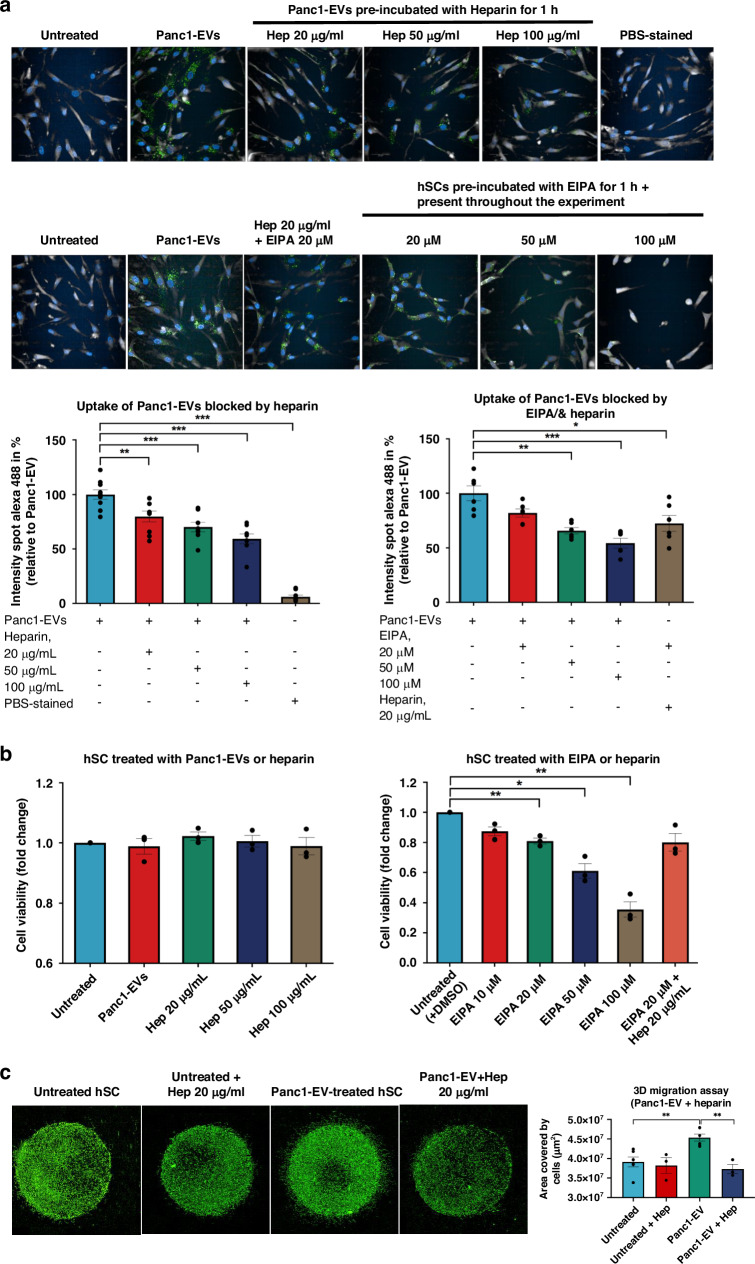
Impact of EV inhibitors on EV-mediated increased migration of hSCs. **a** hSCs were treated with PKH67-labelled Panc1-derived EVs pre-incubated with different concentrations of heparin or EIPA for 1 h or throughout the experiments (24 h). Images were taken by the Operetta CLS^TM^ high-content analysis system. Quantification was performed by measuring the intensity of Alexa Fluor 488. The error bars depict mean ± S.E.M. from three independent experiments. **b** Cell viability of hSCs was measured by PrestoBlue reagent after exposure to Panc1-EVs, heparin or EIPA for 24 h. Data were obtained from three independent experiments and shown as mean ± S.E.M. **c** 3D migration assay of hSCs mixed with Panc1-EVs pre-incubated with heparin for 1 h before embedding in a Matrigel drop and monitored for 4 days. Images (pseudo-colour) were taken by the digital phase contrast function of the Operetta system. Quantification was performed by measuring the area covered by cells (µm^2^). Statistical difference of all data was analysed by two-tailed unpaired Student’s *t*-test. *p < 0.05, **p < 0.01, ***p < 0.001.

To further confirm the role of EVs in SC-increased migration, exogenous addition of EV inhibitors, heparin and EIPA (5-[N-ethyl-N-isopropyl] amiloride), was employed to evaluate if uptake of EVs by hSCs was mediated through surface binding to receptor or/and macropinocytosis, a major route of EV uptake [20]. It has been reported that the heparan sulfate proteoglycans (HPSGs)-dependent uptake route is highly relevant for the internalisation and functional activity of EVs, as HPSGs function as receptors for EVs [21–23]. As for EIPA, an inhibitor for macropinocytosis, it inhibits the uptake of EVs through the blocking of membrane ruffling by decreasing cytosolic pH and inactivation of Rac1 and Cdc42 GTPases [24]. Expectedly, following exposure to Panc1-derived EVs pre-treated with different doses of heparin, the EV uptake by hSCs decreased significantly in a dose-dependent manner. Increasing doses of heparin from 20 μg/mL to 100 μg/mL resulted in a decreased proportion of PKH67-stained EVs by 20.19% ± 4.95 to 40.74% ± 4.49, respectively, as compared to Panc1-derived EV uptake without heparin (Fig. 2a). As for EIPA pre-treatment on the hSCs, although the EV uptake decreased significantly in the presence of 50 μM and 100 μM by 34.26% ± 2.98 and 45.68% ± 4.42, respectively (Fig. 2a), the cell viability of hSCs reduced significantly after treated with EIPA for 24 h by 38.9% ± 4.9 and 64.5% ± 5.2, respectively (Fig. 2b, right), indicating that the EIPA treatment was too toxic for the hSCs. The cell viability of hSCs remained intact in the presence of heparin, regardless of the concentration (Fig. 2b, left). It was also observed that combined heparin (20 μg/mL) and EIPA (20 μM) together did not significantly reduce the uptake of Panc1-EVs (reduction of 27.47%) compared to the single treatment (reduction of 20.19% and 18.05% by heparin 20 μg/mL and EIPA 20 μM, respectively), suggesting that targeting at two different uptake routes of Panc1-EVs in the case of hSCs does not lead to a synergistic effect. Using the concentration of 20 μg/mL of heparin to perform a 3D migration assay, the results show that heparin significantly reduced the area covered by hSCs triggered by Panc1-derived EVs, indicating that the migratory ability of hSCs was inhibited by heparin. These results suggest that Panc1-derived EV uptake by hSCs was partially regulated by cell surface HPSGs and these EVs were, in part, responsible for the migratory effect on hSCs.

### Murine neuroinvasive cancer cell-derived EVs influence SC phenotype by promoting increased migration and are enriched with p75NTR

**Fig. 3:**
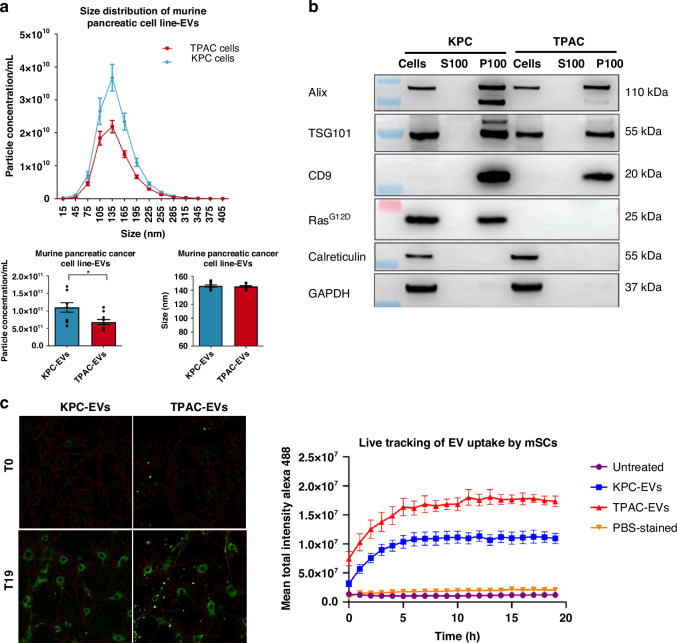

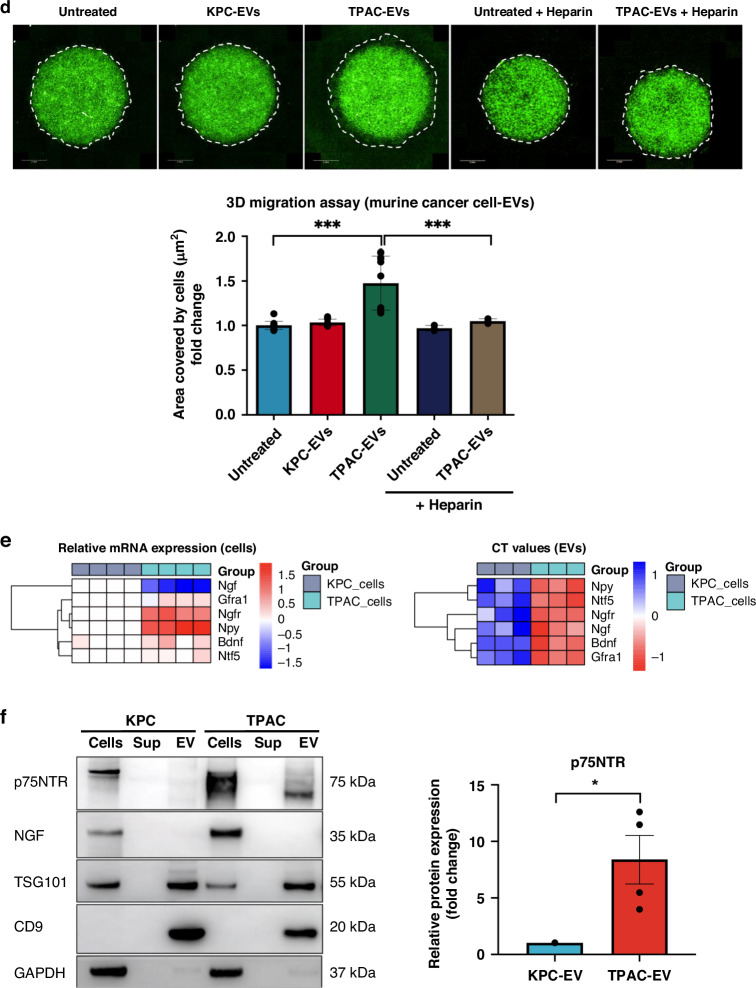
EVs from neuroinvasive cancer cells promote increased migration of mSCs and enriched with p75NTR. **a** NTA analysis of P100 fractions from KPC and TPAC cells. Data represent the mean ± S.E.M. of ten replicates. **b** Western blot analysis of EV and non-EV markers of proteins from whole cell lysates (Cells), EV-depleted supernatant (S100) and EV pellets from pancreatic cell lines (P100). **c** Live-tracking analysis of PKH67-labelled KPC- and TPAC-EV (green fluorescent spots) uptake by hSCs. Representative image of green fluorescent spots accumulated in the cells (red border) at time points 0 and 19. Line graphs illustrated the mean total intensity of Alexa Fluor 488 over 19 h. Measurements and analysis were performed by the Operetta CLS^TM^ high-content analysis system. **d** 3D migration assay of mSCs mixed with EVs from KPC or TPAC cells embedded in a single Matrigel drop and monitored for 4 days. Images (pseudo-colour) were taken by digital phase contrast of the Operetta system. Quantification was performed by measuring the area covered by cells (µm^2^). The data are mean ± S.E.M. from three independent experiments. **e** qRT-PCR analysis of the targets in KPC and TPAC cells as well as their respective EVs. The heatmap (left) depicting neurotrophin-related targets in KPC and TPAC cells was generated based on 2^-ΔΔCT^ relative to KPC cells. The heatmap (right) showing the raw CT values of the targets in KPC- and TPAC-derived EVs. Log of the raw values was used for the generation of a heatmap using R software version 4.1.2. Data were obtained based on four independent replicates. **f** Western blot analysis of p75NTR and NGF expression levels in whole cell lysate (Cells), EV-depleted supernatant (Sup) and EVs (EV) from KPC and TPAC cells. Data were obtained based on four independent replicates and shown as mean ± S.E.M. Statistical difference of all data here was analysed by a two-tailed unpaired Student’s *t*-test. *p < 0.05, **p < 0.01, ***p < 0.001.

To investigate further the underlying mechanisms of increased SC migration, non-neuroinvasive murine cancer cells, KPC, and neuroinvasive murine cancer cells, TPAC that were established previously [14, 25] were employed in this study to perform horizontal 3D coculture assay in the presence or absence of heparin (Supplementary Fig. 6A). As described in the current literature, p75NTR/TrkA/NGF signalling pathway plays an important role in NI [7, 15, 16]. Hence, the lysates from mSCs were probed for p75NTR, TrkA and NGF antibodies. p75NTR expression levels were strongly increased in the mSCs facing TPAC cells compared to those confronted with KPC cells (Supplementary Fig. 6B), which is a profile of dedifferentiated SCs during nerve injury or repair. On top of that, the increased p75NTR levels in the mSCs facing TPAC cells decreased significantly in the presence of heparin (Supplementary Fig. 6B), implying that the increased p75NTR levels may be EV-mediated. Apart from that, EVs collected from the coculture assay show that p75NTR levels in the EVs increased significantly when mSCs confronted with TPAC (TPAC-mSC) compared to TPAC alone and mSCs confronted with KPC cells (KPC-mSC) using Alix as an EV marker for normalisation (Supplementary Fig. 6C), suggesting that the interaction between mSCs and neuroinvasive TPAC cells promoted the enrichment of p75NTR expression in the EVs. To study the effect of EVs from murine cancer cell lines on mSCs, EVs were first isolated by differential ultracentrifugation, characterised by NTA and western blot. As illustrated by the size distribution graph, KPC cells (1.10 × 10^11^ ± 1.35 × 10^10^ particle/mL) secreted significantly higher particle concentration compared to TPAC cells (6.78 × 10^10^ ± 7.23 × 10^9^ particle/mL) while there was no significant difference in terms of the particle size (KPC: 146.2 ± 1.46 nm; TPAC: 145.5 ± 0.98 nm) (Fig. 3a). Similar to the characterisation of EVs from human pancreatic cancer cell lines, Alix, TSG101 and CD9 were detected whereas calreticulin and GAPDH were undetected in the P100 fractions from KPC and TPAC cells after ultracentrifugation (Fig. 3b, Supplementary Fig. 7). Next, live-tracking analysis showed that mSCs took up significantly higher amount of TPAC-EVs than KPC-EVs within the same time frame (endpoint mean total intensity = KPC-EVs: 1.09 × 10^7^ ± 8.76 × 10^5^; TPAC-EVs: 1.73 × 10^7^ ± 9.20 × 10^5^; *p*-value = 0.0005) (Fig. 3c), suggesting the differential EV uptake efficiency in the mSCs. For the functional effect, TPAC-EVs increased the migration of mSCs significantly compared to KPC-EVs, as demonstrated by the 3D migration assay (Fig. 3d). On top of that, the increased migration of mSCs after exposure to TPAC-EVs was reduced significantly in the presence of heparin (20 μg/mL), suggesting the possibility of EV-mediated migration of the mSCs (Fig. 3d).

To explore the EV cargoes from KPC and TPAC cells, several neurotrophic factors including p75NTR and other potential candidates related to NI were examined by qRT-PCR and western blot. As illustrated by the heatmap, the expression levels of *Npy* and *Ngfr* in the TPAC cells were significantly increased compared to the KPC cells whereas *Ngf* is significantly expressed in the KPC cells compared to TPAC cells (Fig. 3e, left). As for the EV levels, since there is currently no universal loading control used for the normalisation of the CT values, the CT values were used for the heatmap illustration. The mean CT values of all the genes in KPC-EVs were above 30 (*Npy* = 33.35 ± 0.76; *Nft5* = 35.26 ± 0.39; *Ngfr* = 35.90 ± 1.84; *Ngf* = 31.09 ± 0.23; *Bdnf* = undetermined, 40 was used for the heatmap generation; *Gfra1* = 33.87 ± 0.18), suggesting that they were lowly expressed in the KPC-EVs. On the other hand, the mean CT values of these genes, especially *Npy, Ngfr* and *Ngf* were significantly lower compared to KPC-EVs (*Npy* = 28.15 ± 0.31; *Nft5* = 31.15 ± 0.26; *Ngfr* = 27.32 ± 0.18; *Ngf* = 29.65 ± 0.18; *Bdnf* = 31.31 ± 0.64; *Gfra1* = 31.65 ± 0.09), suggesting the enrichment of these genes in the TPAC-derived EVs (Fig. 3e, right). Interestingly, the protein levels of p75NTR were 8-fold enriched in the TPAC-derived EVs compared to the KPC-derived EVs, but not its ligand, NGF (Fig. 3f). This is in agreement with another study showing the absence of NGF in the EVs [13], implying the selective packaging of EVs by the cells.

### Tumour tissue- and plasma-derived EVs from patients with Pn1 promote increased SC migration

**Fig. 4:**
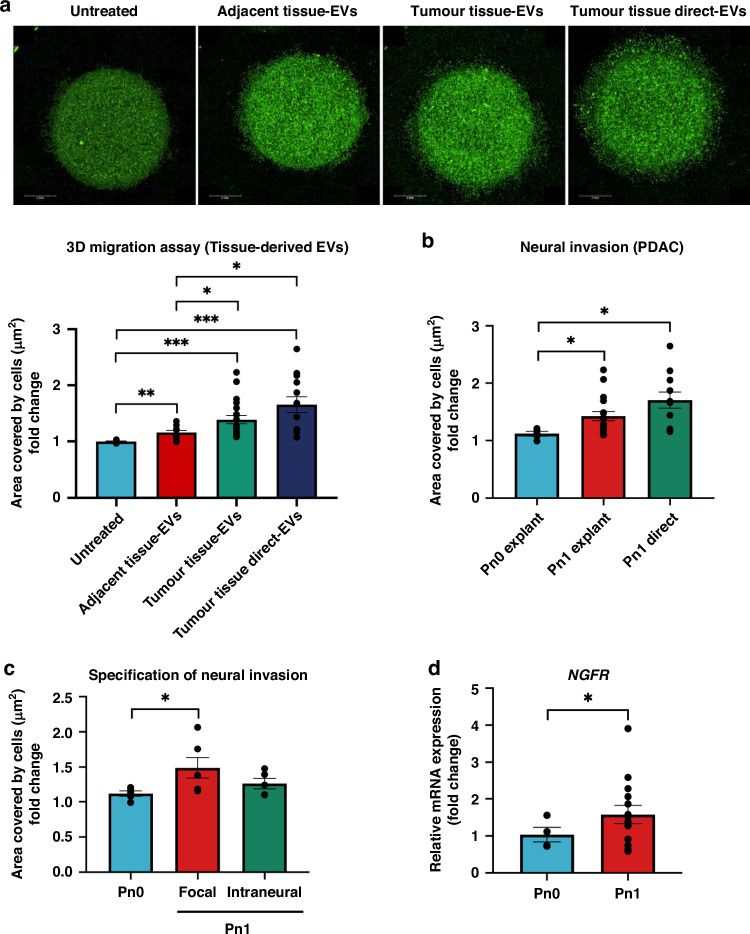

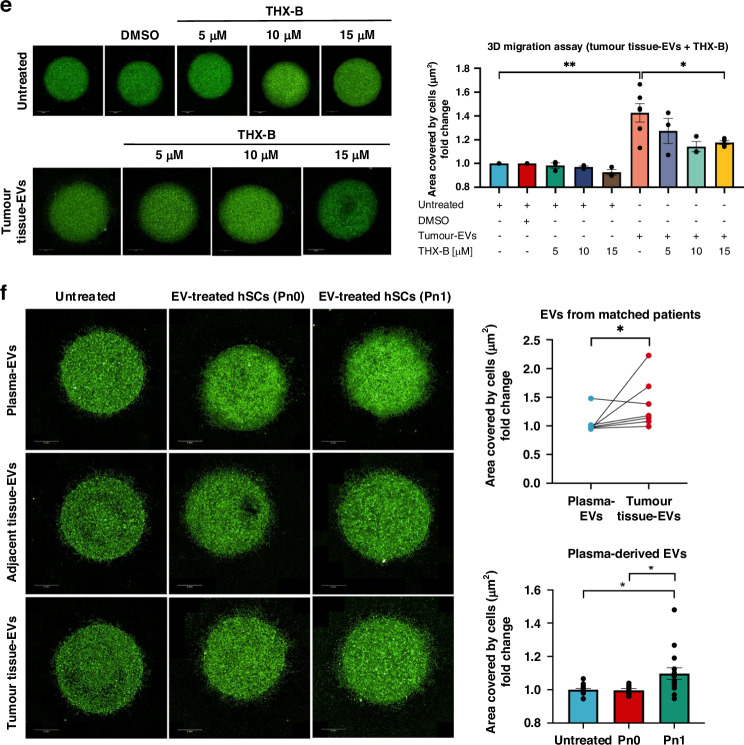
Tumour tissue- and plasma-derived EVs from patients with Pn1 enhance the migratory capacity of hSCs. **a** 3D migration assay of hSCs mixed with EVs from adjacent tissues, tumour tissues and tumour tissue direct embedded in a single Matrigel drop and monitored for 4 days. Images (pseudo-colour) were taken by digital phase contrast of the Operetta CLS^TM^ high-content analysis system. Quantification was performed by measuring the area covered by cells (µm^2^). The error bars depict mean ± S.E.M. Patient number for adjacent normal tissue-EVs = 10; Tumour tissue-EVs = 20; Tumour tissue direct-EVs = 13. **b** Analysis of the results from 3D migration assay in (**a**) hSCs treated with tumour tissue-EVs or tumour tissue direct-EVs from PDAC patients without NI (Pn0) and with NI (Pn1). The error bars depict mean ± S.E.M. *n* for Pn0 = 5; Pn1 explant = 16; Pn1 direct = 11. **c** Analysis of the results from 3D migration assay in (**a**) hSCs treated with tumour tissue-EVs from PDAC patients without NI (Pn0) and with NI (Pn1) categorised by focal NI and intraneural NI. The error bars depict mean ± S.E.M. *n* for Pn0 = 5; Pn1 explant = 16; Pn1 direct = 11. **d** qRT-PCR analysis of *NGFR* expression levels in the hSCs treated with tumour tissue-derived EVs harvested from 3D migration assay shown in (**a**). *n* for Pn0 = 4; Pn1 = 14. **e** 3D migration assay of hSCs mixed with tumour tissues-EVs embedded in a single Matrigel drop cultured in medium containing no THX-B, or 5 μM, 10 μM, 15 μM THX-B for 4 days. Images (pseudo-colour) were taken by digital phase contrast of the Operetta CLS^TM^ high-content analysis system. Quantification was performed by measuring the area covered by cells (µm^2^). The error bars depict mean ± S.E.M. Patient number for tumour tissue-EVs = 6. **f** Representative images of 3D migration assay of hSCs treated with plasma-, adjacent tissue-, and tumour tissue-EVs from matched patients. Statistical difference between hSCs treated with plasma-EVs and tumour tissues-EVs was analysed by Wilcoxon matched-pairs signed rank test. For hSCs treated with plasma-derived EVs from patients with Pn0 (*n* = 8) and Pn1 (*n* = 15), statistical differences were analysed by two-tailed unpaired Student’s *t*-test after passing the normality test by the Shapiro–Wilk method. Statistical differences for (**a**) and (**c**) were analysed by the Mann–Whitney *U*-test. Statistical differences for (**b**), (**d**) and (**e**) were analysed by two-tailed unpaired Student’s *t*-test after passing the normality test by the Shapiro–Wilk method. *p < 0.05, **p < 0.01, ***p < 0.001.

To validate the findings obtained from the murine cancer cell model, human pancreatic tissues were obtained to isolate the EVs from the freshly obtained tissues (direct), adjacent tissues (explant model) and tumour tissues (explant model). As shown by the HE staining, the tissues remained intact after 24 h of incubation (Supplementary Fig. 8A). Although the particle concentration from the adjacent tissues (4.58 × 10^11^ ± 1.05 × 10^11^ particle/mL) and tumour tissues (5.74 × 10^11^ ± 1.01 × 10^11^ particle/mL) did not differ significantly, the mean diameters distribution of the particles from the tumour tissues (157.3 ± 2.58 nm) were significantly larger than those from the adjacent tissues (144.3 ± 2.30 nm) (Supplementary Fig. 8B). TEM images and western blot analysis of EV markers confirmed the identity of EVs, in addition to showing the different subtypes of EVs from different tissues (Supplementary Fig. 8C, D). For the functional effect of these patient tissue-derived EVs, hSCs were treated with the tissue-derived EVs in the 3D migration assay. Expectedly, hSCs increased migration significantly after exposure to the tumour tissue- and tumour tissue direct-EVs compared to untreated and that of the adjacent tissue-EVs (Fig. 4a). In particular, when the patients were categorised based on Pn status in the pathology reports, it could be observed that hSCs increased migration significantly after exposure to the tumour tissue-EVs from the PDAC patients diagnosed with Pn1 compared to those with Pn0 (Fig. 4b). To increase the depth of NI analysis, further specification of NI, i.e tumour focal or circular appear in nerve sheet and tumour intraneural, was analysed with HE and IHC staining of PDAC tissue sections (Supplementary Fig. 9). The tissues sections displaying tumour focal NI secreted EVs that increased the migration of hSCs significantly compared to tumour tissue-derived EVs from patients tissues with Pn0 (Fig. 4c). To study the effect of tumour tissue-EVs on SC phenotype, qRT-PCR was performed with the RNAs from the hSCs treated with tumour tissue-EVs in the 3D migration assay. The results show that the increased hSC migration after exposure to the tumour tissue-EVs from those patients diagnosed with Pn1 also corresponded to a higher expression level of *NGFR* in the hSCs (Fig. 4d), suggesting the potential effect of neuroinvasive EVs on the dedifferentiation of hSCs, thereby increasing their migration ability. Despite a limited number of patients, it was shown that tumour tissue EVs from patients with Pn1 seem to contain higher protein expression levels of p75NTR than those EVs from Pn0 patients (Supplementary Fig. 10), suggesting that p75NTR may be enriched in the neuroinvasive tissue-derived EVs that could influence the SC behaviour. This observation warrants further validation with more patient samples. To verify the possible involvement of p75NTR in the increased migration of hSCs after exposure to tissue-derived EVs, a p75NTR antagonist, THX-B was used in the 3D migration assay. The results show that the increased migration of hSCs in the presence of tumour tissue-EVs (*p*-value = 0.003) reduced significantly with the addition of 15 μM THX-B (*p*-value = 0.023, Fig. 4e), a dose that was not toxic to the hSCs (Supplementary Fig. 11). This result support the hypothesis that tumour tissue-EVs may contain p75NTR that mediates the increased migration of hSCs. To understand the effect of EVs from tumour, peritumoral microenvironment and systemic environment on SC behaviour, hSCs were treated with EVs from PDAC patients with matched adjacent, tumour tissues and plasma in the 3D migration assay (Fig. 4f). Although tumour tissue-EVs increased the migration of hSCs significantly more than plasma-EVs, it should be noted that plasma-EVs from patients with Pn1 did promote stronger migration of hSCs relative to those from Pn0 patients (Fig. 4f). This suggests that PDAC with NI may alter the systemic environment such as distant organs and immune cells to produce EVs that have an impact on SC migration. The characteristics of PDAC patients contributing tissues- and plasma-derived EVs for EV characterisation and 3D migration assay were summarised in Supplementary Table 1.

### Clinical relevance of p75NTR for PDAC patients with NI

**Fig. 5:**
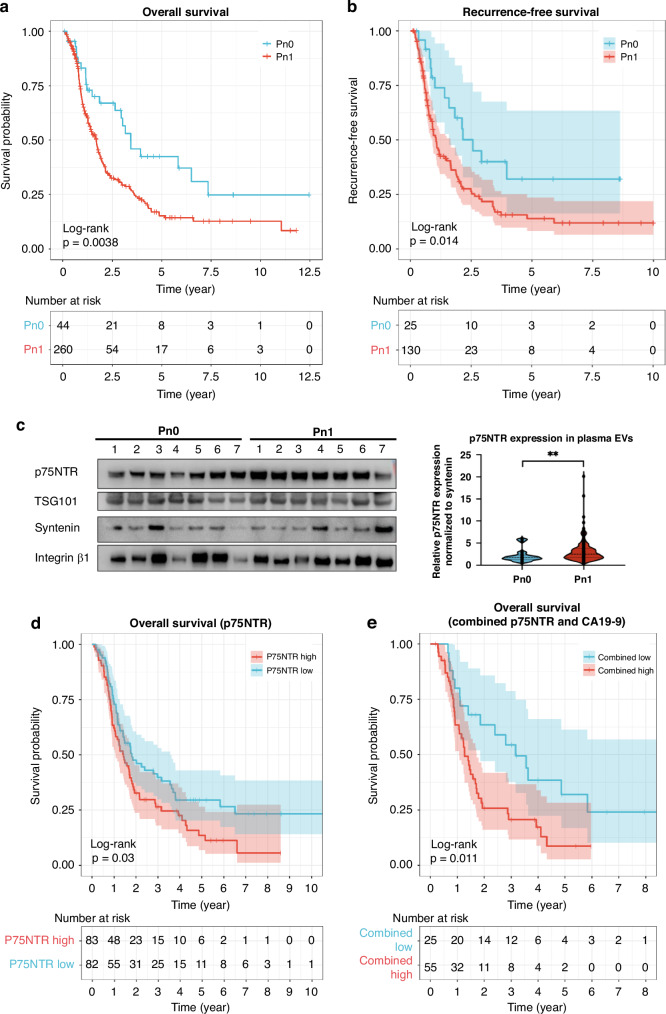
Clinical relevance of p75NTR for PDAC patients with NI. **a**, **b** Kaplan–Meier analyses of OS (**a**) and RFS (**b**) of PDAC patients from University Hospital Dresden based on NI status (Pn0: no NI; Pn1: with NI). **c** Representative samples of western blot analysis of p75NTR and EV markers in plasma-derived EVs from PDAC patients (*n* for Pn0 = 25; Pn1 = 140). Quantification of p75NTR was performed based on the normalisation of syntenin. **d** The prognostic correlation of plasma EV p75NTR from PDAC patients was assessed by Kaplan–Meier analyses for OS. Median cut-off was used to divide the patients into p75NTR high expression and low expression. **e** The prognostic performance of the combination of EV p75NTR and CA19-9 for PDAC patients. Statistical differences for (**a**), (**b**), (**d**) and (**e**) were analysed by Log-rank test with *p* < 0.05 considered as statistically significant. Statistical differences for (**c**) were analysed by the Mann–Whitney *U*-test. *p < 0.05, ***p < 0.001.

While it is accepted that NI is associated with a reduced OS and recurrence-free survival (RFS) for pancreatic cancer patients, there are some publications with opposing results [26, 27]. To confirm the impact of NI on PDAC patient OS and RFS, Kaplan–Meier curve analyses were performed with PDAC patients from UHD based on their Pn status. The patients with Pn1 were significantly correlated with reduced OS and RFS compared to patients with Pn0 (log-rank *p*-value for OS = 0.0038; RFS = 0.014) (Fig. 5a, b). The clinical data of PDAC patients contributing to the OS and RFS analyses were presented in Supplementary Table 2. Since EVs hold great potential as liquid biopsy, it was of high interest to evaluate the clinical relevance of EV p75NTR as a biomarker for PDAC patients with NI. As plasma remains the most accessible source for liquid biopsies and is depleted of platelet-derived EVs, western blot analysis of p75NTR expression in the plasma-derived EVs from a total of 165 PDAC patients (Supplementary Table 3) was performed to evaluate the potential of plasma-derived EV p75NTR for diagnosis and prognosis of NI in patients with PDAC. Since syntenin was demonstrated to be present in plasma-derived EVs ubiquitously [28], the expression levels of p75NTR were normalised to syntenin as an EV marker internal control. p75NTR was significantly higher in the plasma-derived EVs from patients with Pn1 (*n* = 140; mean fold change = 3.186 ± 0.218) compared to Pn0 (*n* = 25; mean fold change = 2.136 ± 0.291) (*p*-value = 0.004) (Fig. 5d and Supplementary Fig. 12). Furthermore, Kaplan–Meier analysis shows that patients with high expression of plasma-derived EV p75NTR displayed a reduced OS (log-rank *p*-value = 0.030) compared to those with low p75NTR expression (Fig. 5e). While the commonly used diagnostic and prognostic biomarker for PDAC, carbohydrate antigen 19-9 (CA19-9) had no significant prognostic ability in the current cohort (Supplementary Fig. 13), the combination of EV p75NTR and CA19-9 demonstrated a superior prognostic performance (log-rank *p*-value = 0.011) than the p75NTR or CA19-9 alone (Fig. 5f), suggesting that the EV p75NTR could complement to the current prognosis of PDAC patients. However, both EV p75NTR and CA19-9 did not show significant prognostic value for RFS in the current study (Supplementary Figs. 13, 14). Based on the univariate and multivariate Cox regression analyses, the EV p75NTR was demonstrated to be an independent prognostic factor (log-rank *p*-value = 0.004) after considering age, gender, tumour stage, grading, resection margin, neoadjuvant therapy, lymphatic invasion, venous invasion and Pn status (Table 1), suggesting that plasma-derived EV p75NTR may be used for risk stratification to predict the prognosis of PDAC patients.

**Table 1:** Univariate and multivariate Cox regression analysis of the EV p75NTR.

Variables		Univariate Cox			Multivariate Cox		
		HR	95% CI	*p*-value	HR	95%CI	*p*-value
Age	*N* = 165	1.00	0.98–1.02	0.747	-	-	-
Sex							
Female/Male	*N* = 165	0.93	0.64–1.35	0.701	-	-	-
Stage							
I/II/III/IV	*N* = 165	1.30	1.06–1.60	0.011	1.25	0.94–1.68	0.129
Grading							
G1/G2/G3	*N* = 149	1.28	0.89–1.84	0.183	-	-	-
Resections							
R0/R1	*N* = 123	1.20	0.66–2.18	0.546	-	-	-
Neoadjuvant chemotherapy							
No/Yes	*N* = 162	1.92	1.17–3.13	0.009	2.15	1.18–3.91	0.012
Lymphatic invasion, L							
No/Yes	*N* = 123	1.89	1.19–2.99	0.007	1.40	0.83–2.35	0.21
Venous invasion, V							
No/Yes	*N* = 123	1.17	0.74–1.87	0.506	-	-	-
Neural invasion, Pn							
No/Yes	*N* = 165	1.59	0.92–2.75	0.096	-	-	-
p75NTR	*N* = 165	1.15	1.06–1.24	0.0004	1.14	1.04–1.24	0.004

## Discussion

EVs are evidently versatile and exist as intercellular mediators for a wide range of normal physiological conditions, but are employed by tumour cells to architect the local and distant microenvironment for tumour progression and metastasis. For instance, tumour exosome-derived migration inhibitory factor (MIF) has been shown to orchestrate the events involved in liver premetastatic niche formation during PDAC metastasis [10]. Furthermore, tumour exosome integrins dictate organ-specific metastasis to the lung, liver or brain and targeting these exosome integrins decreases the metastasis in the respective organs [11]. It is hoped that gaining insight into the specific contribution of tumour-derived EVs to the process of NI could provide opportunities for a deeper understanding of the pathogenesis of NI and therapeutic targeting of NI. While some studies report the role of chemokines and cytokines in promoting pancreatic cancer-associated NI and pain induction [14, 29, 30], the functional implications of EVs on SCs underlying the early steps triggering the invasion of PDAC cells into nerves remain elusive.

The present study demonstrated extensive EV characterisation and elucidation of the role of EVs from multiple sources to prove the role of EVs in altering the phenotype of SC as an early event in the process of NI. Firstly, the EVs from pancreatic cancer cells promote a pro-migratory SC phenotype by triggering dedifferentiation in SCs. In addition to the chemokines and cytokines [29–31], this study additionally shows that EVs represent another group of players involved in the process of NI, highlighting a research niche that was previously unknown. The focus of cancer therapy has gradually shifted to targeting the tumour microenvironment since the last decade, leading to clinical trials depleting the stromal constituents and generation of immunotherapies [32, 33]. It is therefore important to consider the tumour microenvironment in understanding the mechanisms of NI. PSCs form a core cell type of the pancreatic stroma and become activated to be CAFs in response to tumour development. Activated PSCs have been shown to induce neurite outgrowth towards pancreatic cancer cells and contribute to pain generation in PDAC through the secretion of pain factors from DRG [34, 35]. However, the role of PSCs in SC behaviour remains elusive. This study demonstrates that EVs secreted from tPSCs also increased the migration of SCs significantly. The finding is consistent with the current notion that tumour cells reprogramme the PSCs in the microenvironment, resulting in selective packaging of EV cargoes that exert a pro-migratory effect similar to pancreatic cancer cell-derived EVs on SC migration. To further validate this idea, tissue-derived EVs were employed in this study and the results show two-fold implications. Firstly, EV cargoes from the tumour cells and the altered tumour microenvironment, e.g., activated PSCs, tumour endothelial cells and pro-tumoural immune cells have an impact on promoting SC migration, as the tumour tissue-EVs consistently increased the SC migration more than adjacent tissue-EVs. Secondly, this increased migration of SC is more prominently triggered by the tumour EVs from a neuroinvasive environment, as tumour tissue-EVs from patients with Pn1 increased migration significantly than those EVs from Pn0 patients, and it is accompanied by a dedifferentiated phenotype in SC, pointing to a pro-migratory capacity. Collectively, this study reinforces the idea of the vital role of the tumour microenvironment in mediating tumour metastasis, as not only the EVs from pancreatic cancer cells but also the EVs from tPSCs and other components in the microenvironment trigger increased migration of SCs.

This study also attempted to discover the EV cargoes that could be responsible for the SC migration and p75NTR was found to be enriched in the EVs from neuroinvasive cancer cells, tumour tissues and plasma that could be used for risk stratification for the prognosis of PDAC patients. p75NTR, also known as low-affinity neurotrophin receptor or NGF receptor (NGFR), is a single membrane-spanning protein belongs to tumour necrosis factor receptor superfamily [36]. It has recently emerged as a potential therapeutic target for pharmacological control of neurotrophin activity in neurological diseases [37, 38]. In PNS, p75NTR is upregulated in SCs dramatically following nerve injury to promote the regeneration of nerves and its expression is essential in SCs for SC migration and axonal growth during development [39, 40]. In the context of cancer, p75NTR expression is upregulated and correlates with increased invasiveness and poor prognosis of gliomas and melanoma [41, 42]. In particular, melanoma-derived small EVs carrying p75NTR/NGFR can induce lymphangiogenesis and create a premetastatic niche in lymph nodes, promoting melanoma metastasis [42]. Moreover, p75NTR containing EVs was reported to promote invasion of non-invasive glioma cells, indicating its role in invasiveness promotion that may contribute to metastasis eventually [43]. Thus, in our study, it is conceivable that pancreatic cancer cells secrete p75NTR in the EVs that can be transferred to SCs following EV uptake, leading to increased levels of p75NTR in the SCs by promoting their dedifferentiation, thereby activating downstream p75NTR signalling and facilitating cancer cell invasion into nerves. This study also demonstrates the potential use of plasma-derived EV p75NTR in differentiating Pn patients and as an independent prognostic factor for predicting the OS of PDAC patients, a new perspective of EV-derived p75NTR as a plasma-based liquid biopsy that may support clinical implementation. Another study reported the potential of serum-derived EV p75NTR as a diagnostic biomarker to distinguish patients with chronic inflammatory demyelinating polyradiculoneuropathy (CIDP, *n* = 36) from patients with Charcot–Marie-Tooth type 1a (CMT1a, *n* = 39) with 92.1% sensitivity and 95.0% specificity, showing the possibility of EV p75NTR as a biomarker. Although this study has focused on p75NTR, it is undoubtedly that there are other players in the EVs that may pivotal role in mediating the SC-cancer interactions. This exciting research area warrants further investigation.

Despite demonstrating the role of EVs in promoting SC migration, it is unavoidable that this study has its limitations. Although 3D coculture and migration assays were employed, this study nevertheless lacks an in vivo animal model to confirm the different cell type interactions and the effect of EVs particularly on SCs, but not other cell types. A follow-up study with orthotopic transplantation of PDAC cells with inducible *Rab27* knockout may confirm the role of EVs on SC migration, as Rab27 controls the secretion of EVs [44]. Furthermore, there was no detailed proofing of the p75NTR mechanism in the current study, which highlights that much work remains to be done in delineating the detailed mechanism of the transfer of EV p75NTR to SCs. Last but not least, a larger validation cohort comprising PDAC patients with different ethnicity groups and metabolic profiles is required to confirm the clinical potential of plasma-derived EV p75NTR as a biomarker to identify NI and predict the survival of PDAC patients.

In conclusion, this study lays the foundation for the role of EVs in altering the SC behaviour, providing an understanding of how tumour-secreted factors, specifically EVs, orchestrate the early step of the NI process which opens new avenues for early detection, prognosis and therapeutic intervention.

## Supplementary information

Supplementary Information

Movie S1

## Data Availability

All data are available in the main text or the supplementary materials. If not available in the main text or the supplementary materials, they are available from the corresponding authors on reasonable request.
