# Supplementary material for: Extracellular vesicles from pancreatic cancer and its tumour microenvironment promote increased Schwann cell migration
Source: Br J Cancer. 2025 Jan 25;132(4):326–39. doi: 10.1038/s41416-024-02915-0 (PMC11832759; doi:10.1038/s41416-024-02915-0)
Supplement: Supplementary file 1 — Supplementary Information [file 41416_2024_2915_MOESM1_ESM.pdf]

Supplementary Information for

Extracellular vesicles from pancreatic cancer and its tumour microenvironment promote increased Schwann cell migration

Fang Cheng Wong *et al.*

*Corresponding author. Email: Christoph.Kahlert@uniklinikum-dresden.de (C.K.); Email: fangcheng.wong@uniklinikum-dresden.de (F.C.W.)

This PDF file includes:

Supplementary Text

Figs. S1 to S14

Tables S1 to S5

Movies S1

Supplementary Text

Materials and methods

Cell lines and primary cultures

The following cell lines were purchased from American Type Culture Collection (ATCC) and cultured according to the supplier's recommendations: human pancreatic cancer cell line Panc1, MiaPaca2, BxPC3, AsPC1, Capan1 and Hs766T. Panc1, MiaPaca2 and Hs766T were cultured in Dulbecco's Modified Eagle Medium (DMEM) (Gibco, Carlsbad, California, USA) supplemented with 10% fetal calf serum (FCS) (Gibco, Carlsbad, California, USA) and 1% Penicillin/Streptomycin (Sigma-Aldrich, St. Louis, Missouri, USA) at 37°C in a humid atmosphere, saturated with 5% CO_2 while BxPC3 and AsPC1 were cultured in Roswell Park Memorial Institute (RPMI) 1640 medium (Gibco, Carlsbad, California, USA) supplemented with 10% FCS and 1% Penicillin/Streptomycin at 37°C in a humid atmosphere, saturated with 5% CO_2. Capan1 cell line was cultured in Iscove's Modified Dulbecco's Medium (IMDM) (Gibco, Carlsbad, California, USA) supplemented with 10 % FCS and 1% Penicillin/Streptomycin at 37°C in a humid atmosphere, saturated with 5% CO_2. Human pancreatic ductal epithelial cells (HPDE) were a kind gift from Professor Christian Pilarsky (Erlangen, Germany) and cultured in keratinocyte serum-free medium (KSFM) supplemented with human recombinant epidermal growth factor (rEGF) and bovine pituitary extract (BPE) (Gibco, Carlsbad, California, United States). Murine pancreatic cancer cell lines KPC3039 (KPC, derived from KPC mouse model with genotype of $Kras^{+/LSLG12D}$;$Ptf1^{+/CRE}$;$Trp53^{+/fl}$) and TPAC18654 (TPAC, derived from TPAC mouse model with genotype of $Ela\text{-}TGFa^{tg}$;$Ptf1^{+/CRE}$;$Trp53^{fl/fl}$;$p65^{fl/fl}$) were a kind gift from Professor Ihsan Ekin Demir (Munich, Germany) [1,2] and cultured in high glucose DMEM medium (Sigma-Aldrich, St. Louis, Missouri, USA) supplemented with 10% FCS and 1% Penicillin/Streptomycin at 37°C in a humid

atmosphere, saturated with 5% CO_2. Primary human Schwann cells/hSCs derived from human spinal nerve and mouse Schwann cells/mSCs from C57BL/6 were purchased from ScienCell Research Laboratories (Catalog #1700 and #M1700-57, ScienCell Research Laboratories, Carlsbad, USA) and cultured in complete Schwann cell medium containing 5% fetal bovine serum (FBS), Schwann cell growth supplement cocktail and 1% Penicillin/Streptomycin (Catalog number #1701, ScienCell, Carlsbad, USA). Cell lines used in the study were regularly tested for mycoplasma contamination. Pancreatic cancer cell lines were checked for STR profiles in the beginning of the study.

Pancreatic cancer specimen and corresponding adjacent normal tissues were obtained from pancreatic adenocarcinoma patients at the Department of Visceral, Thoracic and Vascular Surgery, University Hospital Carl Gustav Carus, Technische Universität (TU) Dresden, Dresden, Germany with informed consent. The protocol was approved by the local ethics committee of the University Hospital Carl Gustav Carus (Ethikvotum EK499122017). Primary cultures of pancreatic cancer stellate cells (tPSCs) and adjacent normal stellate cells (nPSCs) were isolated from the pancreatic cancer specimen and adjacent normal pancreas tissues, respectively, according to the publication [3]. Briefly, the tissues were cut into pieces (1 mm x1 mm size) and cultured in a mixed medium of DMEM (supplemented with 20% FBS and 1% Antibiotic-Antimycotic) and KSFM (supplemented with rEGF, BPE and 1% primocin (InvivoGen, San Diego, California, United States) in 2:1 ratio for at least 14 days. After the PSCs grew out of the tissues and established, DMEM supplemented with 20% FBS and 1% Antibiotic-Antimycotic was used for culturing. The non-tumorigenic skin fibroblast cell line BJ (purchased from ATCC CRL-2522), nPSCs and tPSCs were cultured in DMEM supplemented with 20% FCS and 1% Antibiotic-Antimycotic (Gibco, Carlsbad, California, United States) at 37^oC with 5% CO_2. Primary human pancreatic stellate

cells/HPaStc isolated from human pancreas were purchased from ScienCell Research Laboratories (Catalog #3830) and cultured in complete Stellate cell medium containing 2% FBS, stellate cell growth supplement cocktail and 1% Penicillin/Streptomycin (Catalog #5301, ScienCell, Carlsbad, USA).

EV isolation from cell lines and human tissues using differential ultracentrifugation

EVs were isolated from the pooled supernatants of six TC-150 cm^2 flasks of Panc1, MiaPaCa2, BxPC3, BJ, HPaStc, nPSCs, tPSCs, KPC and TPAC by differential ultracentrifugation. One day after initial seeding in TC-150 cm^2 flasks, the cells were washed with Phosphate Buffered Saline (PBS, Gibco, Carlsbad, California, United States) and respective medium containing EV-depleted FCS (achieved by ultracentrifugation at 100 000 *x* g for 16 h, 4°C and sterile-filtered with 0.1 μm filter unit) were added to the flasks and incubated for 48 h. After 48 h, the media were first centrifuged at 800 *x* g for 5 min, followed by 2000 *x* g for 10 min to remove dead cells and cell debris. The supernatant was sterile filtered with 0.22 μm filter and subjected to ultracentrifugation at 100 000 *x* g, 2 h, 4°C in an ultracentrifuge (Sorvall MX150+ micro-ultracentrifuge, Thermo Scientific, Darmstadt, Germany). The supernatant was removed and the pellet was washed once with ice-cold PBS, and ultracentrifuged again at 100 000 *x* g, 2 h, 4°C. The resulting pellet was resuspended either in PBS, basal cell culture medium, or RIPA lysis buffer (#9806S, Cell Signalling Technology, Danvers, Massachusetts, United States), depending on the subsequent experiment purposes.

As for EVs from human explant model, the fresh tissues obtained from pancreatic adenocarcinoma patients at the Department of Visceral, Thoracic and Vascular Surgery, University Hospital Carl Gustav Carus, Technische Universität (TU) Dresden, Dresden, Germany (with approval Ethikvotum EK499122017) were cut into slices of 1 mm x 5 mm and cultured in DMEM serum-free medium for 24 h at 37°C with 5% CO_2. The culture medium were then collected and centrifuged at 300 *x* g for 5 min, 2000 *x* g for 10 min and 10 000 *x* g for 30 min before passing through 0.20 μm filter and ultracentrifuged at 100 000 *x* g for 80 min, 4°C. The supernatant was removed and the pellet was washed with cold PBS and subjected to ultracentrifugation of 100 000 *x* g for 120 min, 4°C. The resulted pellet was resuspended in either PBS, SC basal medium or RIPA lysis buffer, depending on the subsequent experiment purpose.

EV isolation from plasma samples using size-exclusion chromatography (SEC) for EV biomarker evaluation

Plasma samples from the enrolled patients were received from the Department of Visceral, Thoracic and Vascular Surgery, UHD, Dresden, Germany after approval by the local ethics committee (EK76032013). Written informed consent from the patients was obtained pre-operatively with the disclosure of research purpose. Approximately 9 mL of blood samples from the recruited patients were collected in an ethylene diamine tetra acetic acid (EDTA)-tube (Sarstedt, Nümbrecht, Germany) on the day of operation or up to a maximum of 10 days before the operation. The blood samples were first centrifuged at 1500 *x* g for 12 min at 4ºC (centrifuge break: 0) and the plasma (upper phase) was transferred to a 15 mL Eppendorf tube for another round of centrifugation at 1500 *x* g for 12 min at 4ºC (centrifuge break: 0). The plasma samples were then aliquoted for storage at -80ºC until use.

For isolation of EVs from plasma samples, SEC columns (IZON Science Ltd, Christchurch, New Zealand) were employed according to the manufacturer's instructions. Briefly, 500 μL of samples were first centrifuged 2,000 *x* g for 10 min and followed by 10,000 *x* g for 30 min before transferring the supernatant to the pre-rinsed SEC columns. Fraction 7-11 were collected as EV-containing fractions and subjected to nanoparticle tracking analysis (NTA) before concentrating to approximately 100 μL with Amicon Ultra-2 Centrifugal Filter Unit (Merck Milipore, Massachusetts, USA). The samples were then proceeded with lysis, protein isolation and quantification according to the procedures in the part of Western blot.

After western blot detection, the results were quantified by Image J (Java-based image processing program developed by National Institutes of Health). The expression of EV p75NTR was normalized to the expression of syntenin. The prognostic correlation of plasma EV p75NTR

from PDAC patients were assessed by Kaplan-Meier analyses for OS (refer to “Patient data analysis” section). While the NI status of the samples was known at the sample collection period, the follow-up information such as OS and RFS were received only at the end of the study.

Negative staining with Transmission Electron Microscopy (TEM)

Fresh EVs resuspended in PBS were used for detection of EV morphology by negative staining with TEM (Electron Microscopy Facility at the Center For Molecular and Cellular Bioengineering, TU Dresden, Germany). Briefly, 10 μL sample was transferred to a grid (300 mesh), incubated for 10 min and followed by washing 2x with water and drying with filter paper. The sample was quickly stained with 1% uranyl acetate/water (UA) for 20 sec and the UA was slowly removed with the tip of filter paper. After complete drying, EVs were imaged with a 100 kv TEM (FEI Morgagni 268D) with a SIS MegaView III camera.

Nanoparticle Tracking Analysis (NTA)

For quantification of the number and size distribution of EVs, the rate of Brownian motion of the particles were determined by ZetaView® nanoparticle tracking analyzer (ZetaView 8.05.05 SP2, Particle Metrix GmbH, Meerbusch, Germany). Before every round of sample measurement, alignment particles (Particle Metrix GmbH, Meerbusch, Germany) with a known average size of 100 nm were used to calibrate the instrument. The pre-acquisition parameters of the instrument were set to a sensitivity of 80 and shutter of 70, whereas post-acquisition parameters were set to a minimum brightness of 20, a maximum size of 200 pixels, and a minimum size of 5 pixels. Samples were diluted in PBS to achieve a particle count in the range of 100 to 200 particles per visual field. EV samples were usually diluted in 1:2000. Samples were loaded to the instrument and analysed based on the videos taken at 11 different positions (30 sec each video) throughout the cell. After

automated analysis of all 11 positions and removal of outlier position if any, the concentration, mean and median diameters of the sample were calculated by the machine software.

Live-tracking of EV uptake by hSCs/mSCs

For fluorescence-labelling of EVs, PKH67 green fluorescent cell linker (Sigma) was used according to manufacturer's instructions. Briefly, EVs diluted with diluent C were mixed with PKH67 dye in diluent C (4 μL in 1 mL diluent C) in 1:1 ratio and incubated for 4 min with gentle shaking. DMEM supplemented with 10% EV-depleted FCS was used to stop the staining reaction. The stained EVs were subjected to ultracentrifugation at 100,000 *x* g, 1:30 h, 4°C. The supernatant was removed while the pellet was washed with PBS and ultracentrifuged again at 100,000 *x* g, 1 h, 4°C. The resulting stained EVs were resuspended in SC basal medium and directly added to the hSCs/mSCs that were seeded on 96-well plate the previous day. Live tracking analysis of PKH67-labelled EV uptake by hSCs was performed by digital phase contrast and AF488 functions of Operetta CLS™ high-content analysis system (PerkinElmer Inc., Waltham, Massachusetts, USA) for 16 h.

Transwell migration assay

For assessing the migratory ability of hSCs after exposure to EVs in a directional manner, transwell migration assay (CytoSelect™ 24-well Cell Migration Assay, Cell Biolabs Inc, San Diego, California, United States) was used according to the manufacturer's instructions ($2.7x10^5$ cells in 300 μl suspension). Briefly, untreated hSCs were placed in the insert of the transwell, whereas respective pancreatic cell line-derived EVs ($1x10^4$ particles per cell) were added to the bottom of the wells for chemoattraction analysis. After 24 h incubation, samples from both setups were washed, stained with Cell Stain Solution giving green fluorescence signal according to the

manufacturer's instructions and imaged by EVOS FL Auto Imaging Systems (Life Technologies, California, United States).

EV inhibitors treatment

For heparin treatment, various source of EVs were pre-incubated with the indicated concentration of heparin, i.e. 20–100 μg/mL at room temperature (RT) on a shaker for 1 h. Simultaneously, the pre-seeded cells were also pre-treated with the same concentration of heparin for 1 h in an incubator with 37°C and 5% CO_2. As for EIPA, only the cells were pre-treated with different concentration of EIPA (20-100 μM) for 1 h and the same concentration of EIPA was present in the EV treatment medium throughout the experiments.

Quantitative real-time PCR

For quantification of the mRNA levels of respective targets of interest, total RNAs from the treated hSCs were extracted with RNeasy Mini kit containing DNase (QIAGEN, Maryland, USA) using QIAcube robotic workstation (QIAGEN, Maryland, USA). RNA concentration was determined using NanoVue Plus Spectrophotometer (GE Healthcare, Illinois, USA) and 500 ng of cellular total RNA was reverse-transcribed using High Capacity cDNA Reverse Transcription Kit (Applied Biosystems, California, USA) to synthesize cDNAs using peqSTAR thermocycler (Peqlab, Germany). After cDNA synthesis, quantitative real-time PCR (qRT-PCR) was performed with primers of 3 pmol/μl (Supplementary Table 4) together with Power SYBR green PCR Master Mix (Applied Biosystems, Warrington, United Kingdom) on a real-time PCR detection system (Applied Biosystems Step One Plus analyser, California, USA). Target gene mRNA levels were normalized against the housekeeping GAPDH mRNA levels, and the analysis was conducted using the ΔΔCt method calculating the rate of induction as $2^{(\Delta\text{Ct ref. gene treated/target gene treated}) - (\Delta\text{Ct ref. gene untreated/target gene untreated})}$.

Western blot

Whole-cell lysates, supernatant or EV pellets were lysed in RIPA lysis buffer (Cell Signalling Technology, Danvers, Massachusetts, United States) complemented with 100X Halt™ protease and phosphatase inhibitor single use cocktail (Thermo Scientific, Darmstadt, Germany) for 30 min on ice and centrifuged at 10,000 *x* g for 30 min, 4ºC to remove insoluble materials. Protein concentration of the samples was determined by the Pierce™ BCA Protein Assay Kit (ThermoFischer Scientific, Rockford, USA) according to manufacturer's instructions and measured the absorbance at 562 nm using microplate reader (Varioskan LUX Multimode Microplate Reader, Thermo Scientific, Darmstadt, Germany). BSA standard curve with a range of 0 to 2000 µg/ml was used to calculate the concentration of the samples. Cell lysates were then mixed in NuPAGE 4X LDS sample buffer dye (Invitrogen, Carlsbad, California, USA) and NuPAGE 10X sample reducing agent (Life Technologies, Carlsbad, California, USA) before denaturing at 90ºC for 10 min and subjecting to SDS-PAGE polyacrylamide gel electrophoresis (4-12% Bis-Tris gel, Life Technologies, Carlsbad, California, USA). Gels were blotted to nitrocellulose membrane (Amersham Biosciences, Uppsala, Sweden) for 70 min at 30 V, stained with ponceau (Sigma-Aldrich, Hamburg, Germany), blocked in 5% non-fat milk (AppliChem GmBH, Darmstadt, Germany) in 1X TBST (TBS containing 0.1% Tween-20) for 1 h at RT and incubated overnight at 4ºC with primary antibodies diluted in 5% BSA. The detailed information of primary antibodies used in the study were listed in Supplementary Table 5. The membranes were washed 3x with 1X TBST, each for 10 min, and were incubated for 1 h at RT with respective secondary antibodies (Anti-rabbit IgG, HRP-linked Antibody #7074 or Anti-mouse IgG, HRP-linked Antibody #7076, Cell Signaling Technology, Massachusetts, USA, 1:1000 dilution). Membranes were then washed 3x with 1X TBST for 10 min each. Bands were visualized with

Immobilon Western Chemiluminescent HRP substrate (Milipore, Darmstadt, Germany) using Fusion FX7 imaging system (Vilber, Marne-la-Vallée cedex 3, France) and analysed with Image J software (Java-based image processing program developed by National Institutes of Health) for quantification.

3D migration assay with THX-B treatment

After hSCs ($1x10^5$ cells) mixed with tumour tissue-derived EVs ($1x10^4$ particles per cell) in a ratio of 1:1 (25 µl Matrigel containing cells + 25 µl of SC basal medium containing EVs) embedded in a single Matrigel drop (in 24-well plate) and incubated for 30 min at 37°C incubator for polymerization, 5 µM, 10 µM and 15 µM THX-B were added to the respective wells containing 1 mL SC basal medium and the same amount of tumour tissue-derived EVs. The gel drops were monitored for a total of 4 days with half of the medium exchanged for fresh medium containing EVs and the same dose of THX-B after 2 days of initial seeding. Images were taken by digital phase contrast of Operetta CLS™ high-content analysis system (PerkinElmer Inc., Waltham, Massachusetts, USA). Quantification was performed by using Harmony software provided by PerkinElmer Inc. to measure the area covered by cells (μm^2). Briefly, the analysis sequence involved detecting cells (Find Nuclei), training to get the region of interest (Find Texture Regions; Texture A = positive cell area; Texture B = negative cell area) and expressed the region covered by cells in area (μm^2).

Patient data analysis

Kaplan-Meier curves used to estimate the OS and RFS of PDAC patients were performed by R software version 4.1.2 ('survival' and 'survminer' packages). The clinical endpoint examined in this study was OS, which was defined as the time of surgery to the time of death or last follow-

up. Patients with OS shorter than 30 days were excluded from the analysis to avoid cases of death due to surgical complications. The expression levels of plasma EV p75NTR obtained from the western blot analysis were first assigned into high and low levels based on the median cut-off value generated by 'surv_cutpoint' function in the 'survminer' R package. The independent prognostic effect of plasma EV p75NTR and other clinical parameters on patient OS were assessed by univariate and multivariate Cox regression analyses by calculating hazard ratio (HR) and 95% confidence interval (CI). An HR <1 indicates decreased hazard for the survival probability in the high-expression group compared with the low-expression group, and *vice versa*.

Statistical analysis

Results concerning cell lines were analysed with unpaired two-tailed Student's *t*-test, whereas the experiments involving patient samples were analyzed either by unpaired two-tailed Student's *t*-test or two-tailed Mann-Whitney U test after testing with Shapiro-Wilk method to determine the data normality. Statistical significance for Kaplan-Meier curves was performed by Log-rank test. Data are expressed as mean $\pm$ S.E.M. and differences were considered statistically significant at the level of p-values < 0.05.

Cell viability analysis

Anti-proliferative effect of EVs or inhibitors on hSCs were examined using PrestoBlue (PB) fluorometric assay based on the ability of metabolically active cells containing mitochondrial reductase enzymes to reduce dark blue resazurin-based compound to red-fluorescent reduced form, which causes a shift in its fluorescence. The excitation and emission wavelength used for this assay are 560 nm and 590 nm, respectively. Briefly, cells were seeded in 96-well plates at 5000 cells/well for 24 h before exposure to the indicative EVs or inhibitors (heparin: 20-100 μg/mL; EIPA: 10-100 μM) for 24 h or up to 72 h. The plate was briefly centrifuged at 500 *x* g for 1 min, the medium

was removed and washed with PBS before the addition of the respective treatment. The treatment were prepared in SC basal medium only and replenished every day. Wells containing SC basal medium only without cells were used as a blank while cells receiving no treatment of any EVs or inhibitors but with respective concentration of dimethyl sulfoxide (DMSO) were served as control. After the indicative period of treatment, 10 µl PrestoBlue Cell Viability reagent (Invitrogen by Thermo Fisher Scientific, Oregon, USA) was added to each well and incubated for 2 h before the measurement of fluorescence signals using microplate reader (Varioskan LUX Multimode Microplate Reader, ThermoFisher Scientific, Darmstadt, Germany).

Cell viability was calculated based on the following equations:

$$\text{Cell Viability (\%)} = \left(\frac{\text{Fluorescence reading of sample} - \text{Fluorescence reading of blank}}{\text{Fluorescence reading of negative control} - \text{Fluorescence reading of blank}}\right) \text{x } 100\%$$

All the experiments were plated in three replicate wells and were performed for three times. Results are expressed as mean ±S.E.M. and presented as cell viability in percentage. Statistical analyses were performed by an unpaired two-tailed Student's *t*-test. $p<0.05$ was considered statistically significant.

Hematoxylin and eosin (HE) and Immunohistochemistry (IHC)

For HE staining, tissue sections from pancreatic cancer and adjacent normal pancreas tissues (4 µm thickness) were dewaxed and rehydrated by successive immersion in xylene (10 min), isopropanol (5 min), 96% ethanol (2 min), 70% ethanol (2 min) and distilled water (dH_2O) (2 min). Samples were stained by hematoxylin for 2 min 30 sec, rinsed by dipping 3 times each in dH_2O and diluted hydrochloric acid (HCl)/dH_2O followed by running warm tap water for 10 min. As counter-staining, samples were stained with eosin for 2 min. Samples were subsequently dehydrated by successive immersion in 70% ethanol (30 sec), 96% ethanol (1 min), isopropanol

(2 min), xylene (5 min) and additional xylene (5 min). After a brief air drying, samples were covered with entellan and a cover slip (24 x 60 mm, Paul Marienfeld GmbH & Co. KG, Lauda-Königshofen, Germany).

For IHC staining, tissue sections were dewaxed and rehydrated by successive immersion in xylene (2 x 5 min), absolute ethanol (3 min), 96% ethanol (3 min), 85% ethanol (3 min), 70% ethanol (3 min) and 1X TBST (10 min). To quench endogenous peroxidases, slices were treated with 1% H_2O_2 (Merck KGaA, Darmstadt, Germany) in 1X TBST solution for 20 min. Antigen retrieval was performed by treating sections with 0.01 M sodium citrate (pH 6) and covered with saran wrap before subjected to microwave at 100% power (540 W) for 5 min. The sections were cooled down for 5 min, proceeded to another round of microwave treatment at 50% power (250 W) for 10 min and cooled down for 30 min. After cooling down, sections were kept in 1X TBST for 10 min and encircled with hydrophobic pen (Dako, Carpinteria, California, United States). For blocking, 100-200 µL of 5% goat block were added per slice and incubated for 60 min at room temperature in a humidified chamber. The blocking solution was removed slowly by paper towel. Primary antibody (p75NTR, Cell Signaling Technology, #8238) was diluted in 1% BSA and incubated overnight at 4°C in a humidified chamber. The next day, sections were rinsed 2x with 1X TBST, each for 10 min. 2 drops of signal stain boost IHC detection reagent (HRP-rabbit, Cell Signaling Technology, #8114; HRP-mouse, Cell Signaling Technology, #8125) were added to each slice and incubated for 30 min at room temperature in a humidified chamber. The sections were washed with 1X TBST for 10 min. Signal detection was performed using 3'3'-diaminobenzidin-tetrahydrochlorid (DAB)-treatment (Vectorlabs, SK-4105) resulting in a brown staining. 1 drop chromogen concentrate was diluted in 1 mL diluent and 200 µL were added per slice. Sections were stained for 30 sec and up to 3 min, while observing an appropriate level of

staining to background. After detection, sections were subsequently washed in running tap water for 5 min, counterstained with hematoxylin for 3 min, rinsed by dipping 3 times each in dH_2O and diluted HCl/dH_2O. Blueing was performed under running warm tap water for 10 min. Samples were subsequently dehydrated by successive immersion in 70% ethanol (3 min), 85% ethanol (3 min), 96% ethanol (3 min), absolute ethanol (3 min), xylene (5 min) and additional xylene (5 min). After a brief air drying, samples were covered with entellan and a cover slip (24 x 60 mm, Paul Marienfeld GmbH & Co. KG, Lauda-Königshofen, Germany) until imaging by Pannoramic Scan II (3DHistech Ltd, Budapest, Hungary).

Immunofluorescence

For immunofluorescence staining of hSCs after treatment of PKH67-labelled EVs, the cells were first washed thrice with ice-cold PBS, fixed in 4% formaldehyde for 10 min and washed twice with PBS. 50 mM NH_4CI was used to quench fixative for 10 min at 37°C. The cells were washed twice with PBS and 0.2% Triton X-100 was added to the cells and incubated for 15 min at room temperature (RT). Blocking was performed with blocking buffer (1% bovine serum albumin + 0.1% Triton X-100 in PBS) for 1 h at RT. Primary antibodies anti-rabbit p75NTR (#07-476, Merck, Kenilworth, New Jersey, United States, 1:100 dilution) were added for overnight at 4°C. The cells were washed thrice with PBS and secondary antibodies Alexa Fluor 594 donkey anti-rabbit (#406418, Biolegend, San Diego, California, United States, 1:200 dilution) were added for 1 h at RT. After washing for three times in PBS, 1 μg/mL DAPI (Carl Roth, Karsruhe, Germany) was added for 10 min at RT and washed once with PBS, before mounting with Immu-mount reagent (Fisher Diagnostics, Schwerte, Germany) and a glass cover slip (24 x 60 mm, Paul Marienfeld GmbH & Co. KG, Lauda-Königshofen, Germany). The immunofluorescence-labelled

tissues were then examined and analysed by laser scanning confocal microscopy (LSM 700, Zeiss, Oberkochen, Germany).

Supplementary figures

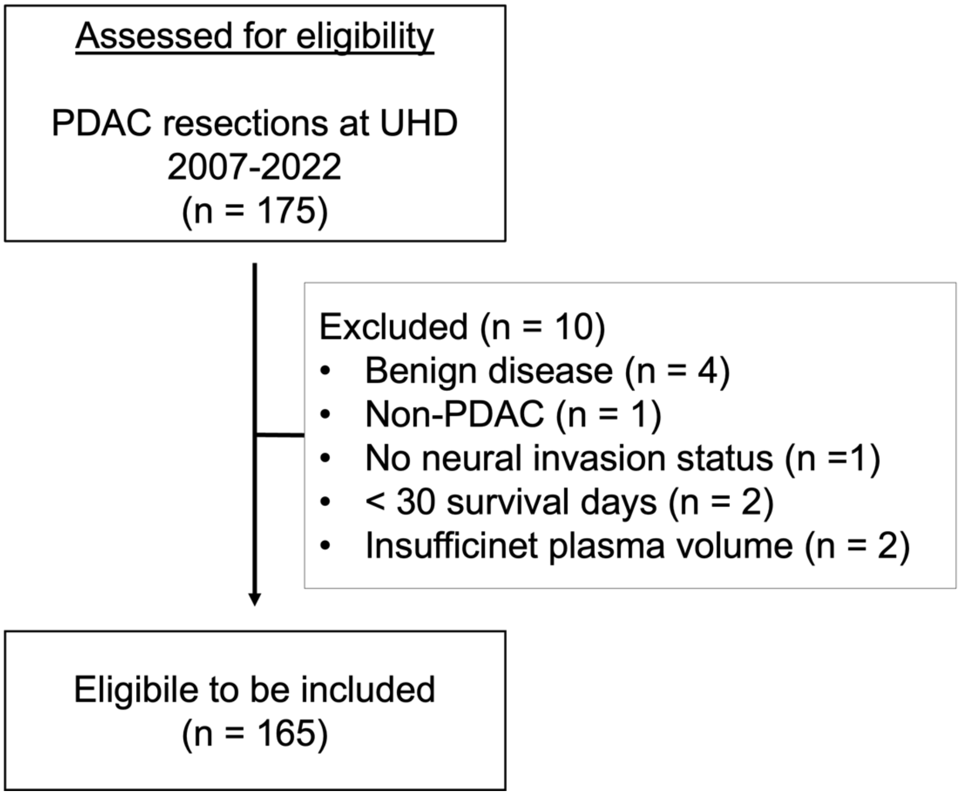

Fig. S1. Schematic flow chart of the selection of patients for plasma-derived EV biomarker evaluation in this study. The number of patients and exclusion criteria for the cohort in this study were clearly indicated in the diagram.

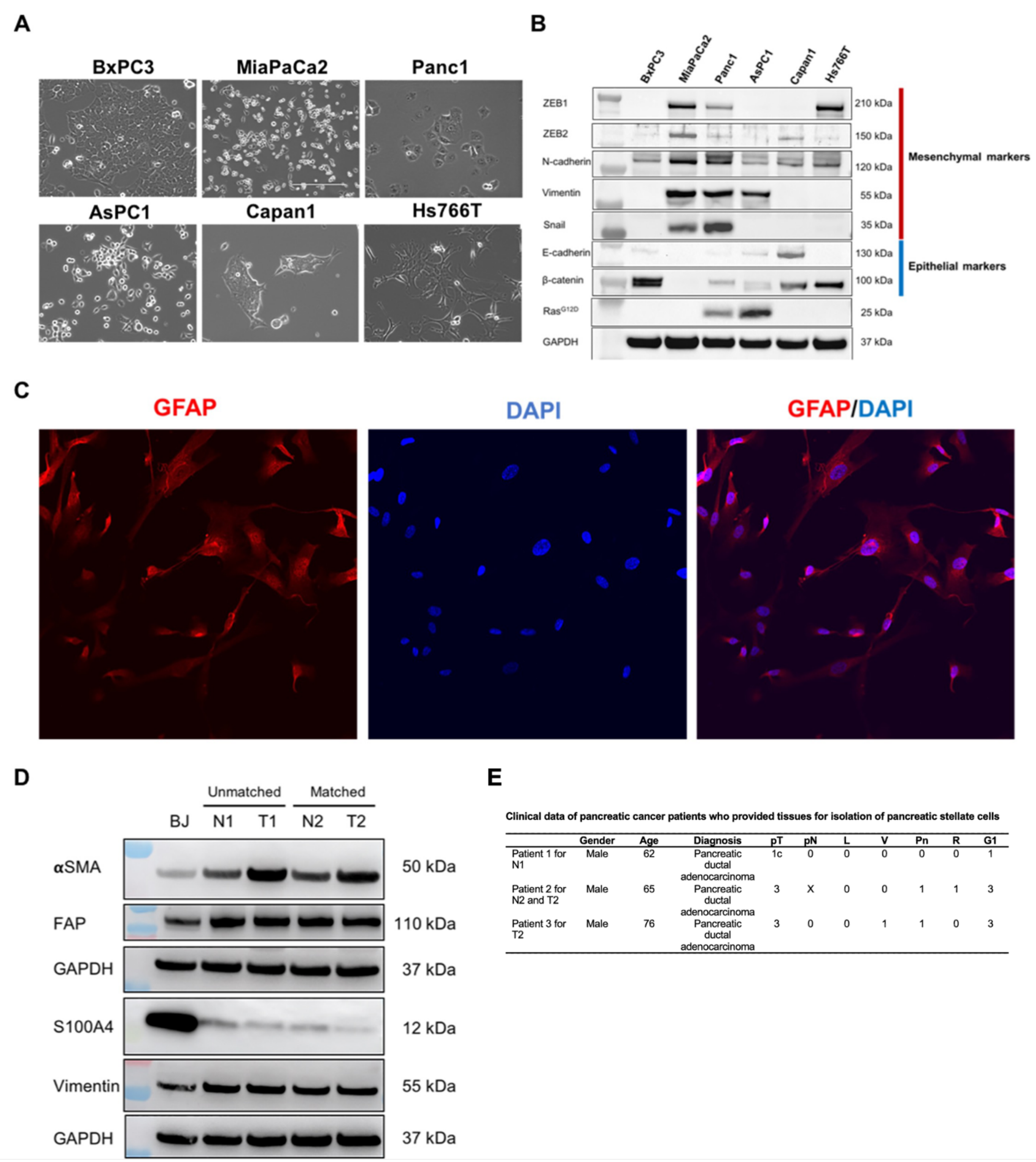

	Gender	Age	Diagnosis	pT	pN	L	V	Pn	R	G1
Patient 1 for N1	Male	62	Pancreatic ductal adenocarcinoma	1c	0	0	0	0	0	1
Patient 2 for N2 and T2	Male	65	Pancreatic ductal adenocarcinoma	3	X	0	0	1	1	3
Patient 3 for T2	Male	76	Pancreatic ductal adenocarcinoma	3	0	0	1	1	0	3

Fig. S2. Characterization of human pancreatic cancer cells, human Schwann cells (hSCs), fibroblasts and pancreatic stellate cells (PSCs). (**A**) Morphological images of six different human pancreatic cancer cell lines. BxPC3, MiaPaCa2 and Panc1 cell lines were derived from primary pancreatic tumour whereas the rest of the cell lines were derived from secondary

metastatic sites (AsPC1: ascites; Capan1: liver; Hs766T: lymph node). Images were taken 72-96 h post-seeding by EVOS FL Auto Imaging Systems (Life Technologies, California, United States). Scale bar denotes 400 μm taken by 10x magnification objective. (**B**) Western blot analysis of epithelial and mesenchymal markers in the whole cell lysates of the six human pancreatic cancer cell lines. (**C**) Immunofluorescence staining of the key marker for hSCs, GFAP (Glial fibrillary acidic protein). Cells were counterstained by nuclei stain, DAPI (4′,6-diamidino-2-phenylindole). Images were taken with LSM ZEISS 880 confocal microscopy (ZEISS, Oberkochen, Germany), 20x objective. (**D**) Western blot analysis of fibroblasts-related markers in the whole cell lysates of the skin fibroblast cell line (BJ), PSCs derived from human adjacent normal pancreatic tissues (N1 = patient 1; N2 = patient 2) and tumour tissues (T1 = patient 1; T2 = patient 2). (**E**) Clinical data of pancreatic cancer patients who provided adjacent normal tissues and tumour tissues for isolation of PSCs used in (D) and Figure 1G (PSC-derived EVs). pT: Pathologic tumour; pN: Pathologic regional lymph node; L: Lymphatic invasion; V: Venous invasion; Pn: Neural invasion; R: Resection; G: Grading.

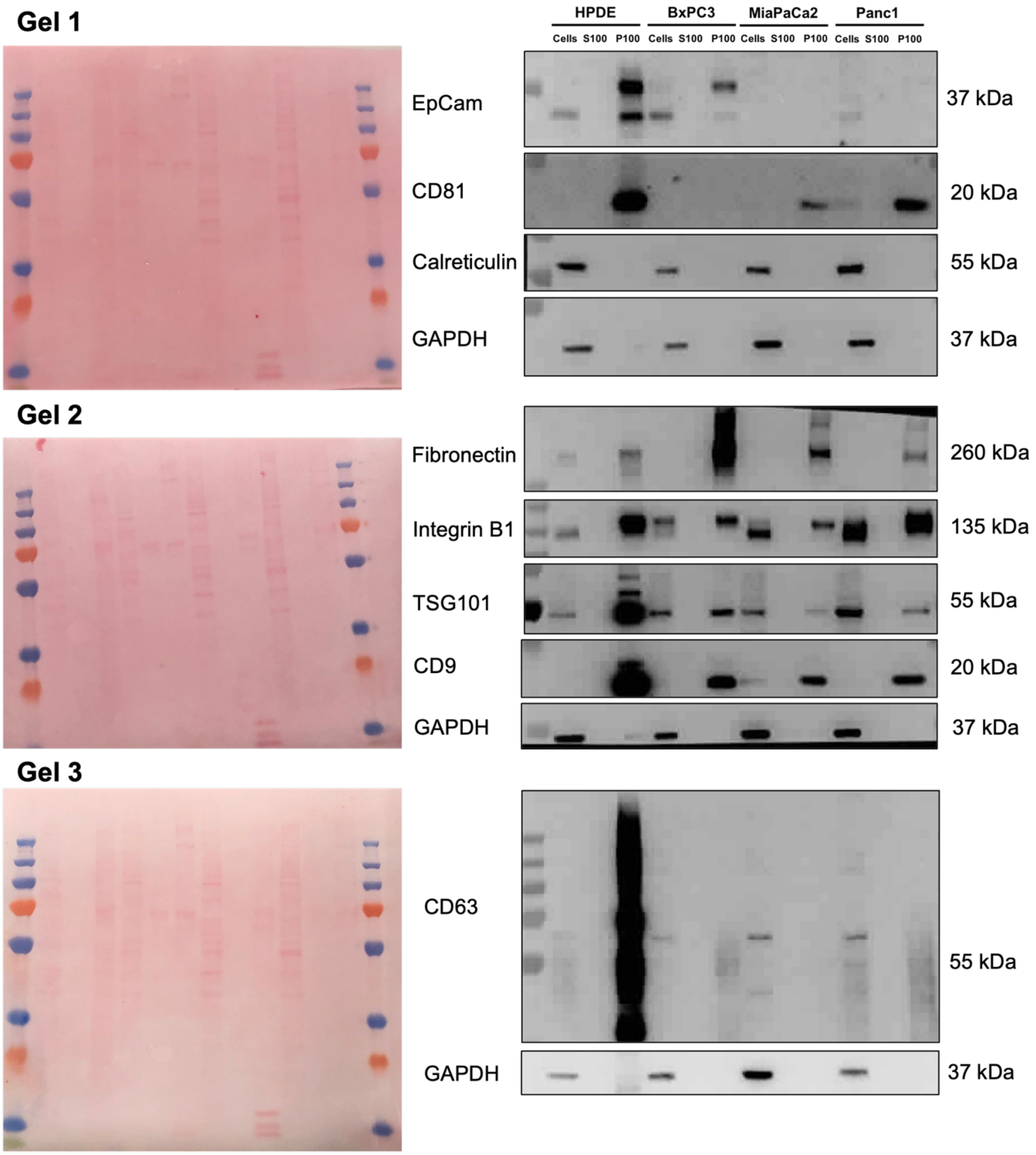

Fig. S3. Ponceau stain of membranes for Figure 1C (human pancreatic cell line-derived EVs).

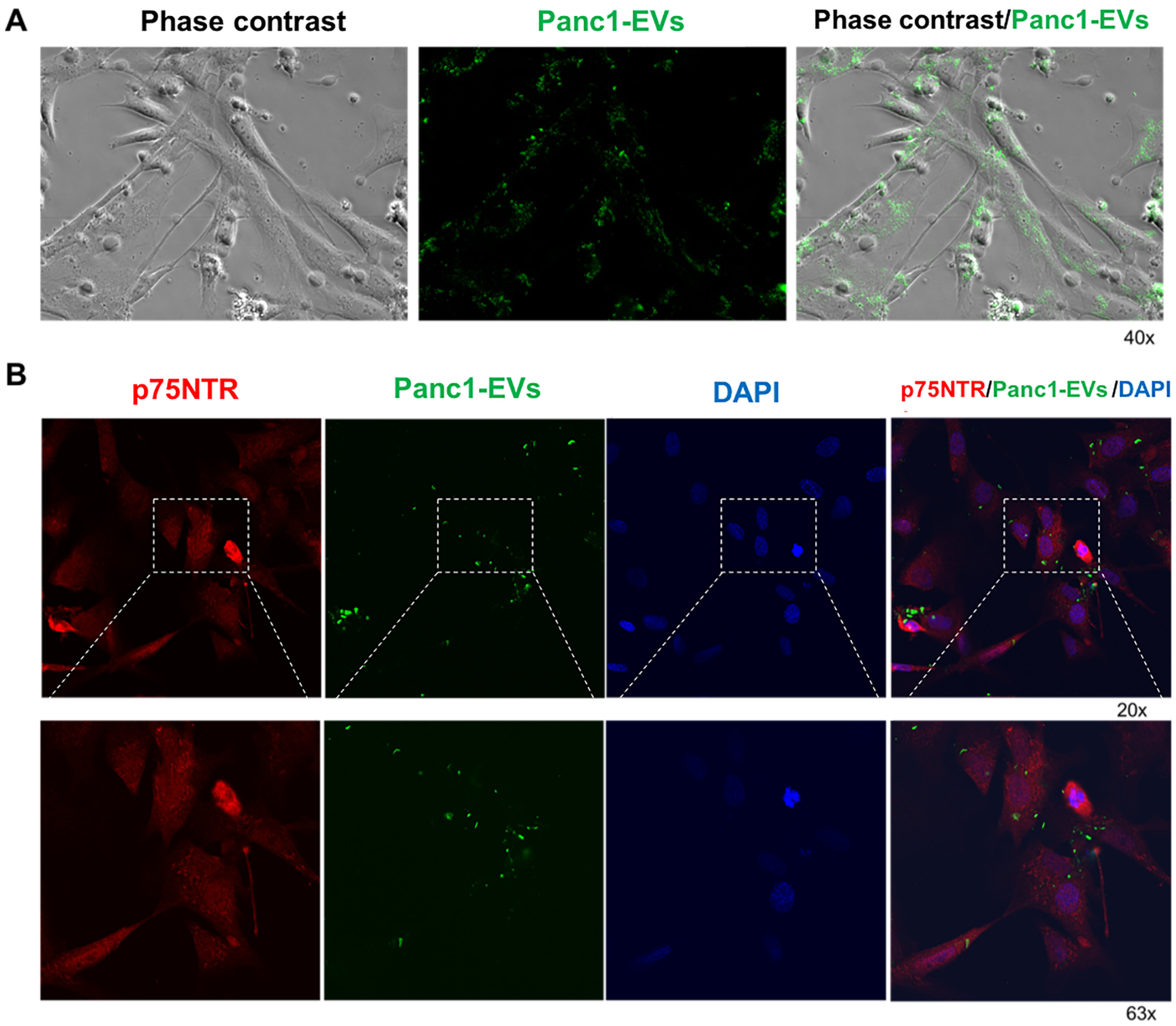

Fig. S4. Uptake of Panc1-EVs by hSCs. (**A**) Phase contrast images of PKH67-labelled Panc1-EVs taken by hSCs. Images were taken 24 h post treatment by EVOS FL Auto Imaging Systems (Life Technologies, California, USA) with 40x objective. (**B**) Immunofluorescence staining of hSCs with anti-P75NTR after exposure to PKH67-labelled Panc1-derived EVs (green fluorescent dots) for 24 h. Cells were counterstained by nuclei stain, DAPI (4′,6-diamidino-2-phenylindole). Images were taken with LSM ZEISS 880 confocal microscopy (ZEISS, Oberkochen, Germany), 20x and 63x objectives. Two independent experiments were performed with three technical replicates per group per experiment.

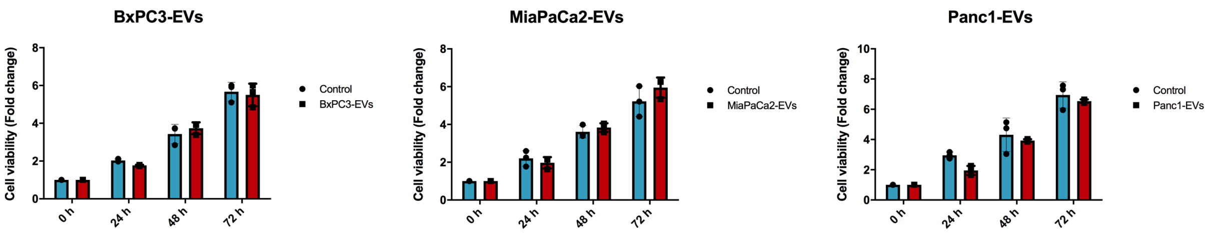

Fig. S5. Cell viability of hSCs after exposure to human pancreatic cancer-derived EVs. Cell viability assay was performed with PrestoBlue reagent after exposure to the indicated cell line-derived EVs for 24 h, 48 h, and 72 h. Measurements of absorbance values were done with VarioScan LUX (Thermo Fischer Scientific, Massachusetts, United States). Data were obtained from three independent experiments and shown as mean $\pm$ S.E.M.

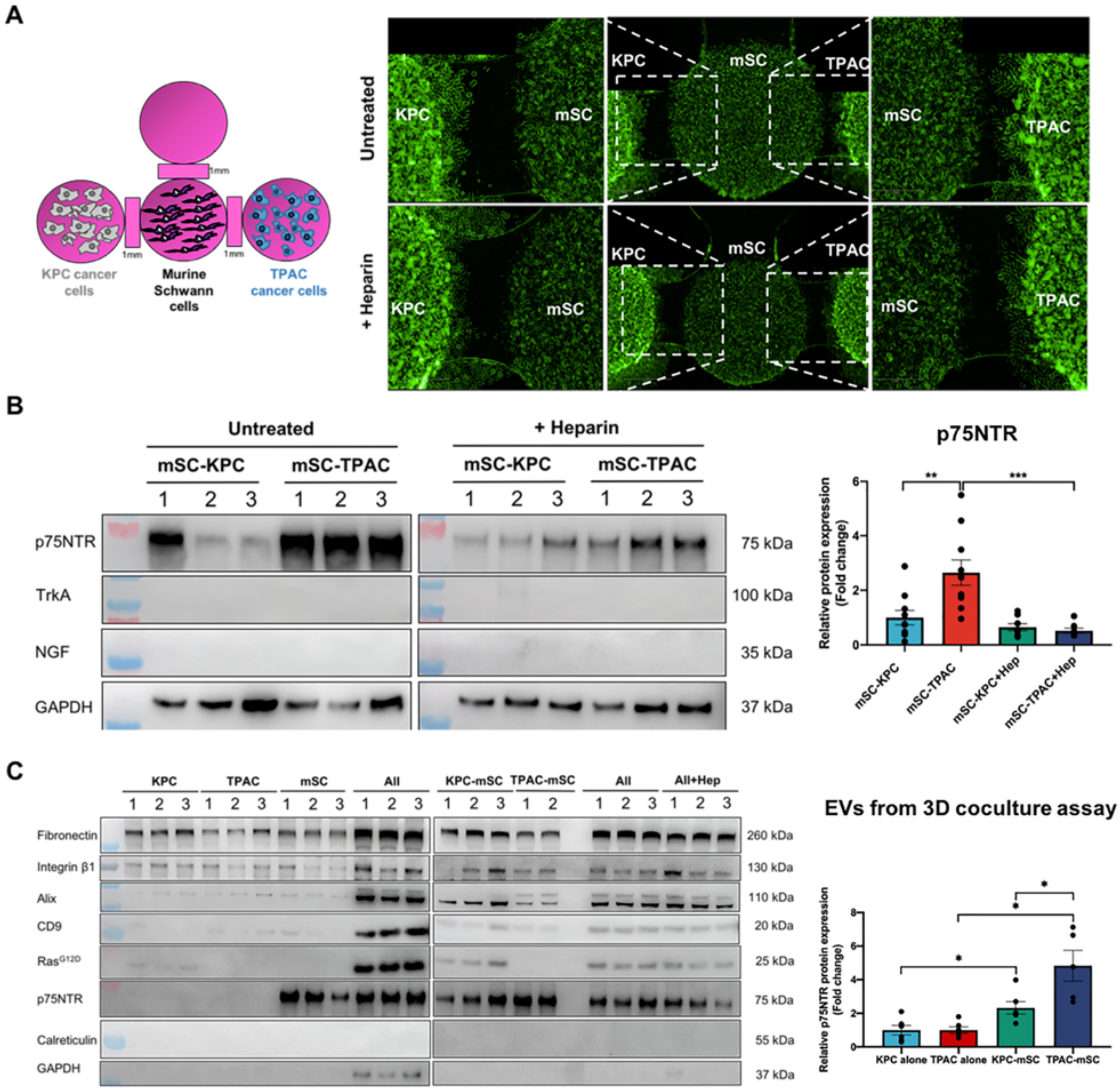

Fig. S6. Murine SCs (mSCs) migrate towards neuro-invasive PDAC cell line and are associated with an up-regulation of p75NTR. (**A**) 3D coculture assay of murine SCs (mSCs) simultaneously confronted to non-neuroinvasive murine pancreatic cancer cells, KPC cells on the left side while neuroinvasive murine pancreatic cancer cells, TPAC cells on the right side. Images were taken by Operetta CLS^{TM} high-content analysis system (pseudo-colour) after 3 days of coculture. Three independent experiments were performed with three technical replicates per

group per experiment. (**B**) Western blot analysis of mSCs harvested from the 3D coculture assay in (**A**). Data were obtained from two independent experiments were performed with three technical replicates per group per experiment and shown as mean ± S.E.M. (**C**) Western blot analysis of EVs harvested from the 3D coculture assay in (A) and other conditions in the same format of the 3D coculture assay (KPC: KPC cells alone; TPAC: TPAC cells alone; mSC: mSCs alone; KPC-mSC: mSCs confronted to KPC only; TPAC-mSC: mSCs confronted to TPAC only; All: mSCs confronted to KPC on the left side and TPAC cells on the right side; All+Hep: mSCs confronted to KPC on the left side and TPAC cells on the right side treated with heparin). Protein levels of p75NTR were normalized to Alix as EV marker control and shown relative to the KPC alone or TPAC alone (normalized at 1). Data were obtained from two independent experiments performed with three technical replicates per group per experiment and shown as mean ± S.E.M. Statistical difference of all data here was analyzed by two-tail unpaired Student's *t*-test. * $p<0.05$. ** $p<0.01$, *** $p<0.001$.

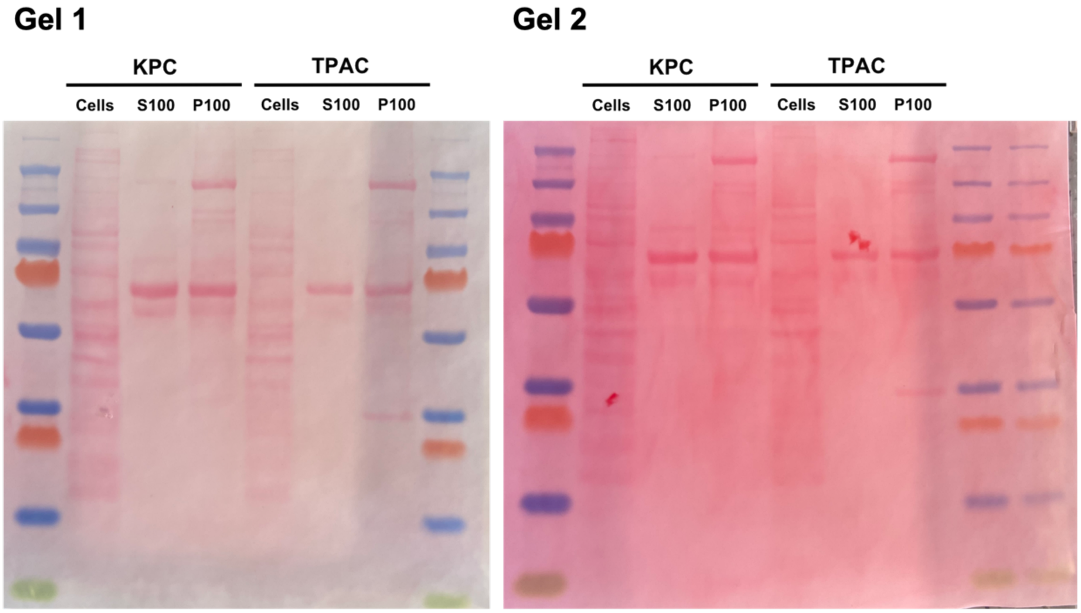

Fig. S7. Ponceau stain of membranes for Figure 3B (murine pancreatic cancer cell lines-derived EVs). The samples for Figure 3F were the same cell lines with the same amount of proteins, 10 μg, but in different passage number.

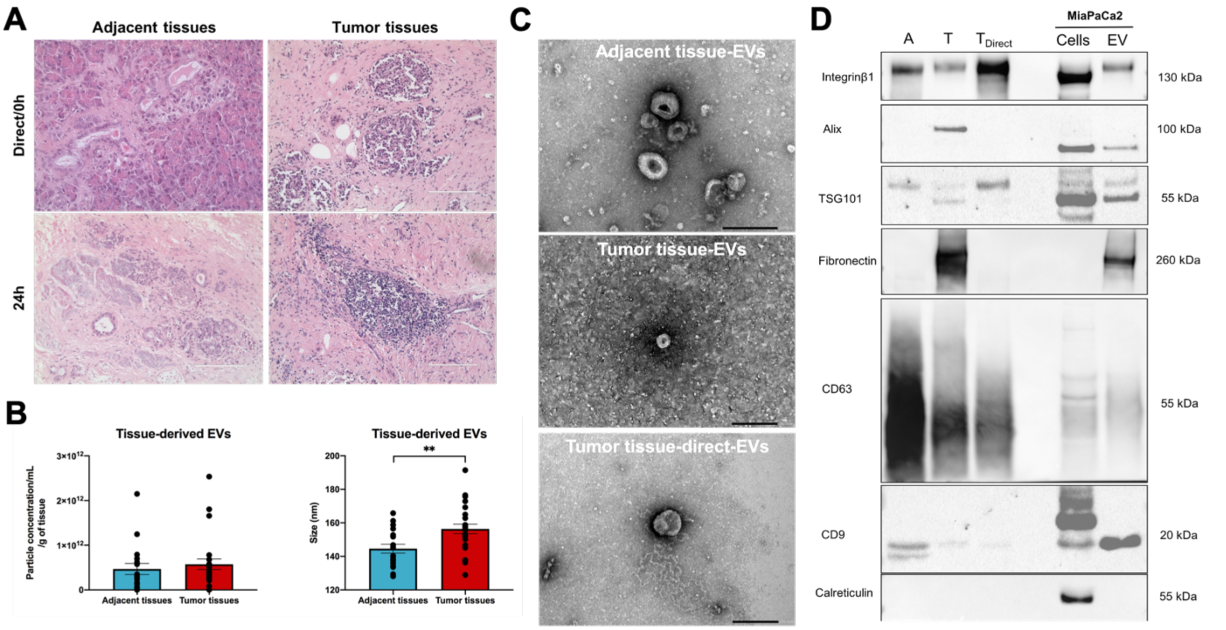

Fig. S8. Characterization of human patient tissues and EVs derived from human tissues. (**A**) Representative images of HE staining of pancreatic tumour tissues and the adjacent tissues at the time of collection (0 h/direct) and after 24 h of serum-free medium incubation (explant model). **(B)** NTA analysis of EVs from pancreatic adjacent and tumour tissues. Data shown were the particle concentration/mL per g of tissues (as the tissues collected from every patient were in different weight) (left) and the size of particles (right). The error bars depict mean $\pm$ S.E.M. (Adjacent tissues, n = 18; Tumour tissues, n = 25). Statistical differences were analysed by two-tailed unpaired Student's *t*-test after passing the normality test by Shapiro-Wilk method. ** p<0.01. **(C)** Representative negative-stain TEM images of EVs from adjacent, tumour and tumour tissue-direct. Scale bar at the bottom right corner of the image indicates 200 nm. **(D)** Western blot analysis of EV and non-EV markers of proteins from adjacent tissues (A), tumour tissues (T) and tumour tissue-direct (T_{direct}). Whole cell lysates and EVs from MiaPaCa2 cell line were used as positive control for this western blot.

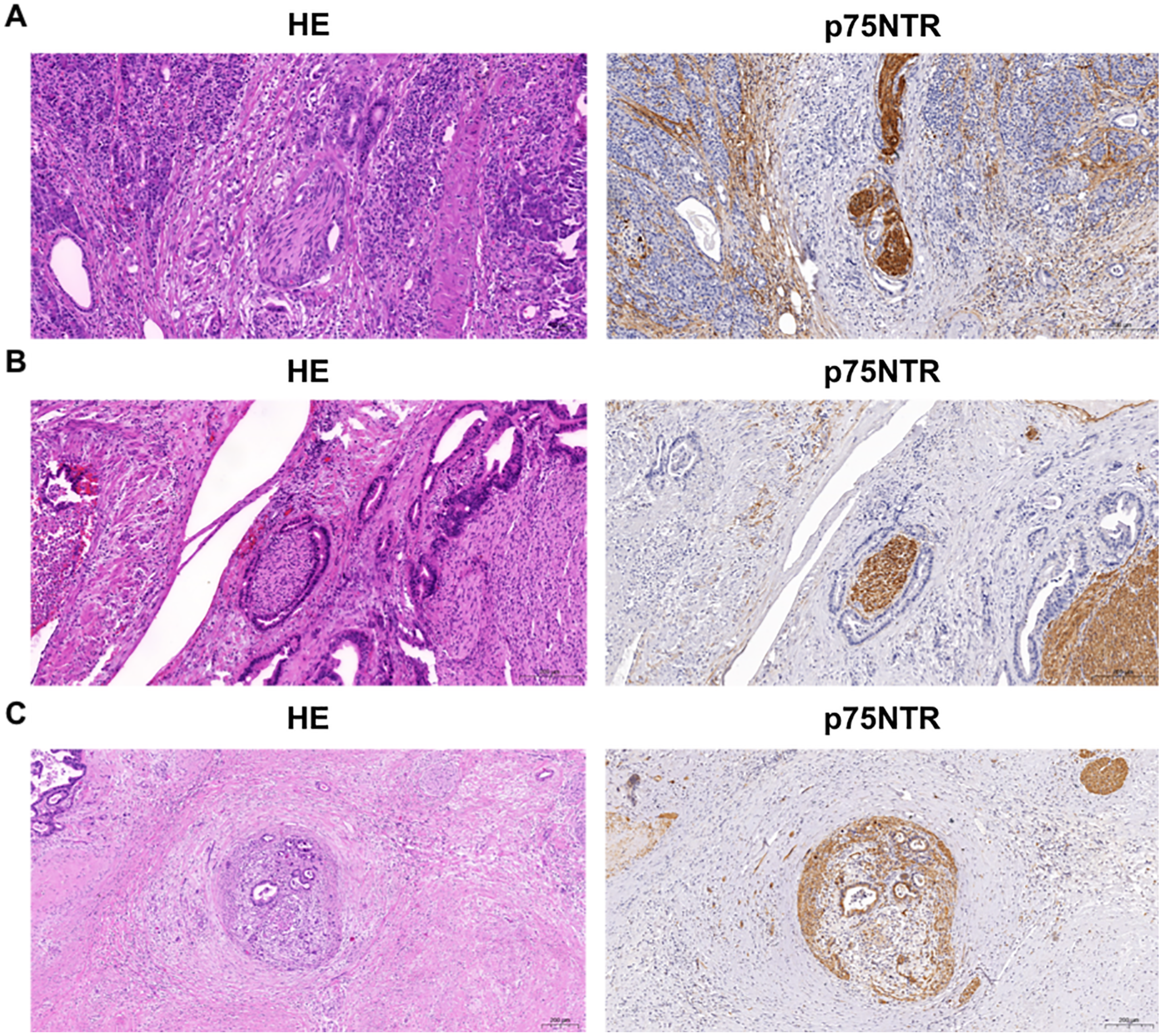

Fig. S9. Specification of neural invasion (NI). Representative HE and IHC images showing different specification of NI (**A**) HE and p75NTR, tumour focal in nerve sheet; (**B**) HE and p75, tumour circular in nerve sheet; (**C**) HE and p75NTR, tumour intraneural.

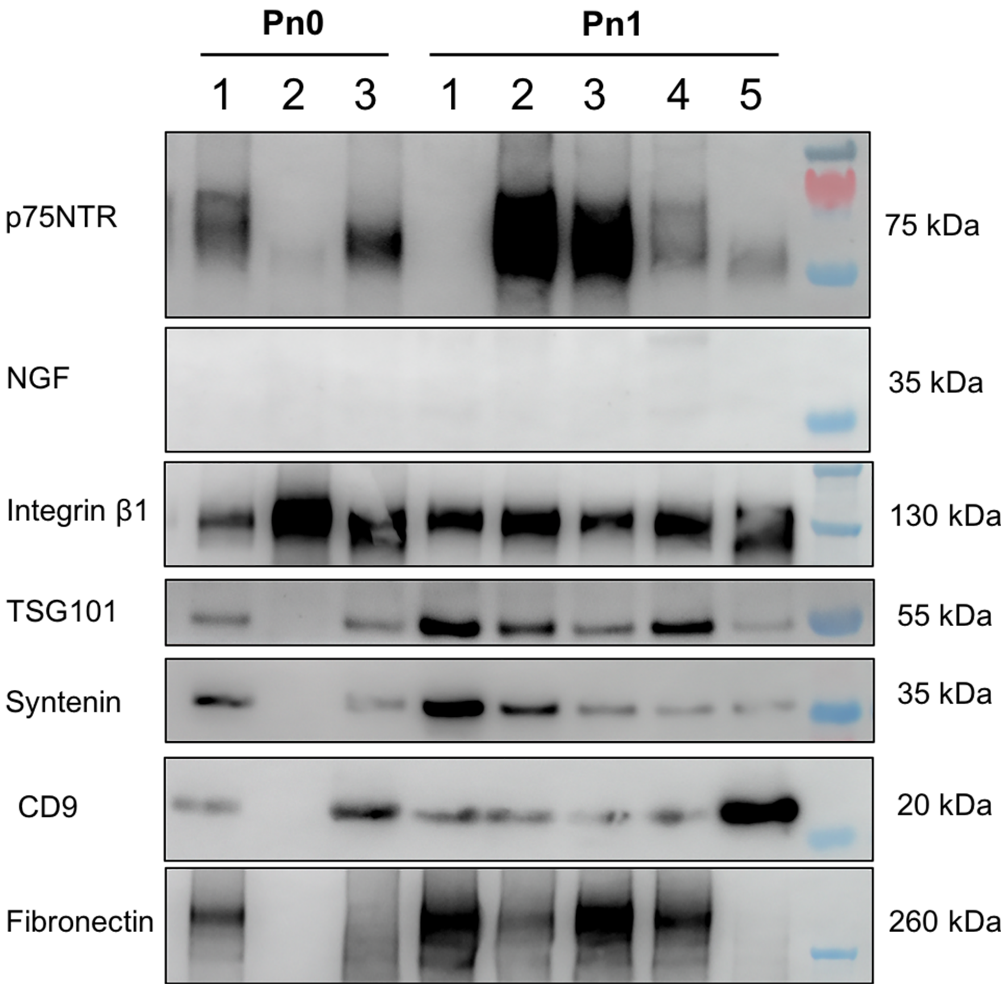

Fig. S10. p75NTR expression levels in the tumour tissue-derived EVs from PDAC patients with and without NI. Western blot analysis of p75NTR, its commonly ligand, NGF, and various EV markers in the EVs from the PDAC patient tumour tissues (explant model). Pn0 indicates patients with no NI (n = 3 patients) and Pn1 represents patients with NI (n = 5 patients).

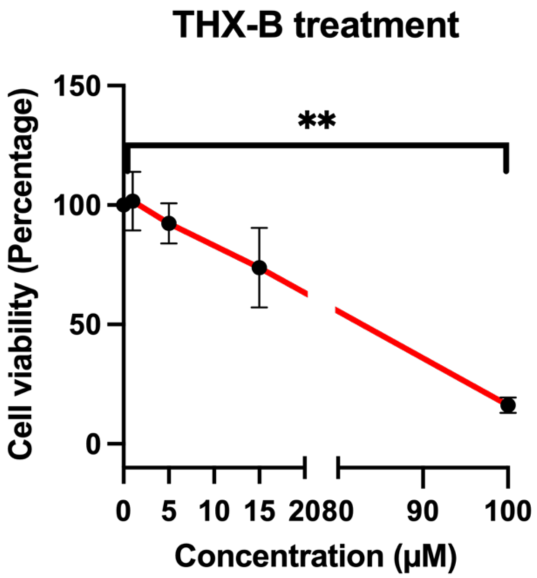

Fig. S11. Cell viability of hSCs after exposure to THX-B. Cell viability assay was performed with PrestoBlue reagent after exposure to different concentration of THX-B (1µM, 5µM, 15µM and 100µM) for 4 days. Measurements of absorbance values were done with VarioScan LUX (Thermo Fischer Scientific, Massachusetts, United States). Data were obtained from three independent experiments and shown as mean + S.E.M.

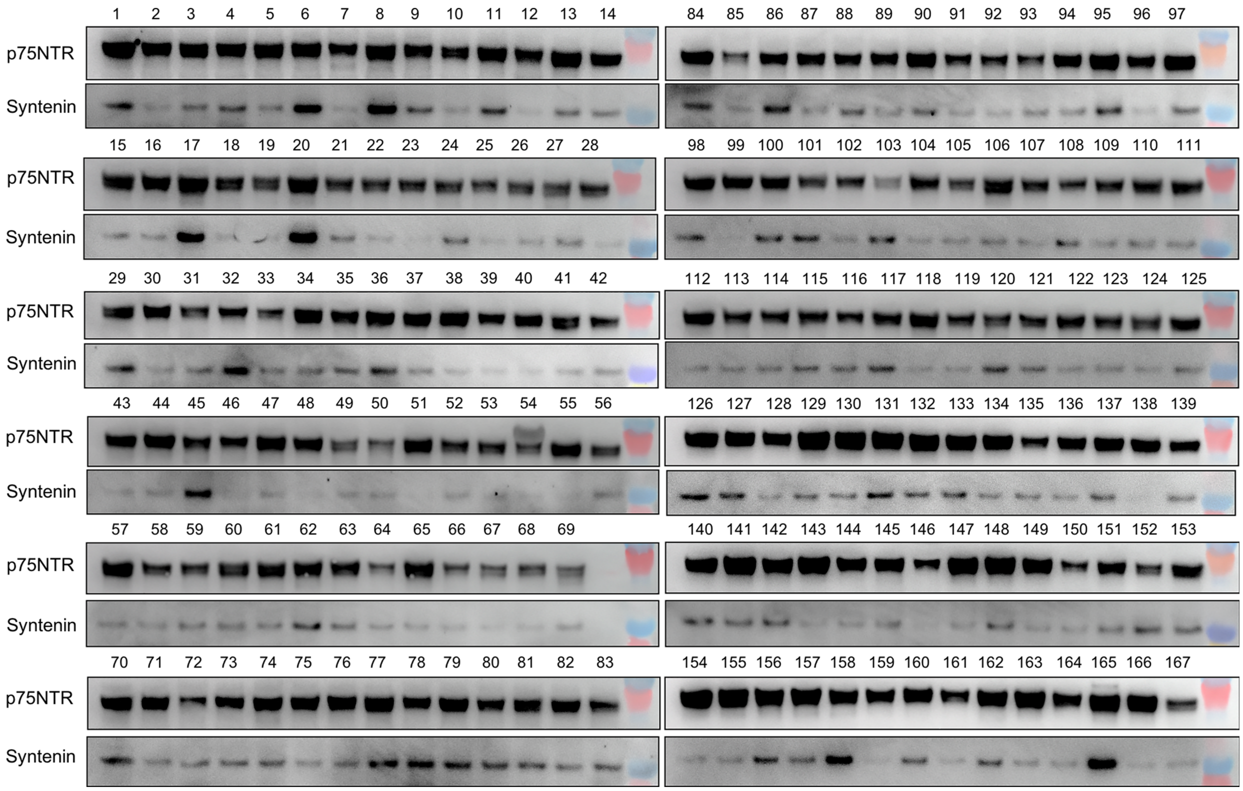

Fig. S12. p75NTR expression levels in the plasma-derived EVs from PDAC patients with and without NI. Western blot analysis of p75NTR and EV marker, syntenin in the EVs from the PDAC patient plasma samples. Pn0 indicates patients with no NI (n = 25 patients; Patient number 1-4, 15-18, 29-32, 43-44, 47, 70-73, 84-87, 100, 102) and Pn1 represents patients with NI (n = 140 patients; the rest of the patient number). Two patients (98, 101) with OS shorter than 30 days were excluded at the later stage when OS data were received during analysis stage.

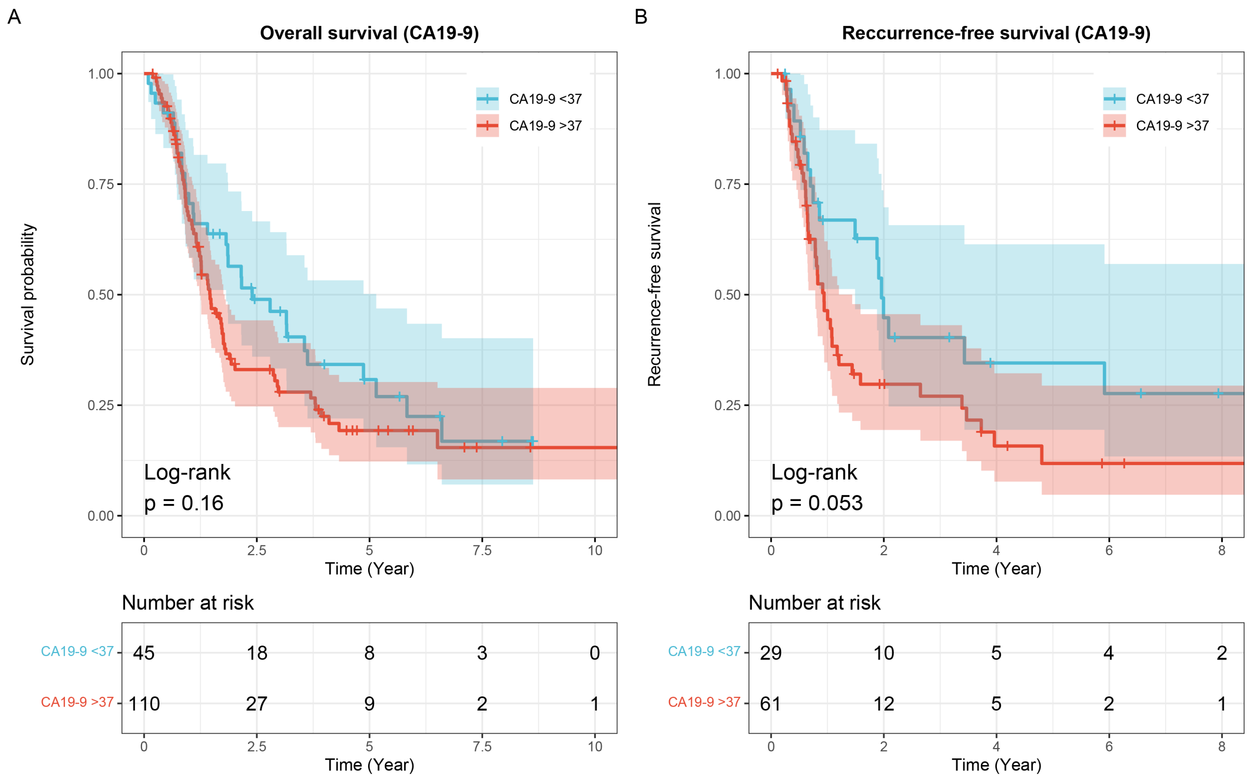

Fig. S13. The prognostic value of serum CA19-9 for PDAC patients. The commonly routine used cut-off value of 37 U/mL was employed in this study for OS (**A**) and RFS analyses (**B**). CA19-9: Carbohydrate antigen 19-9.

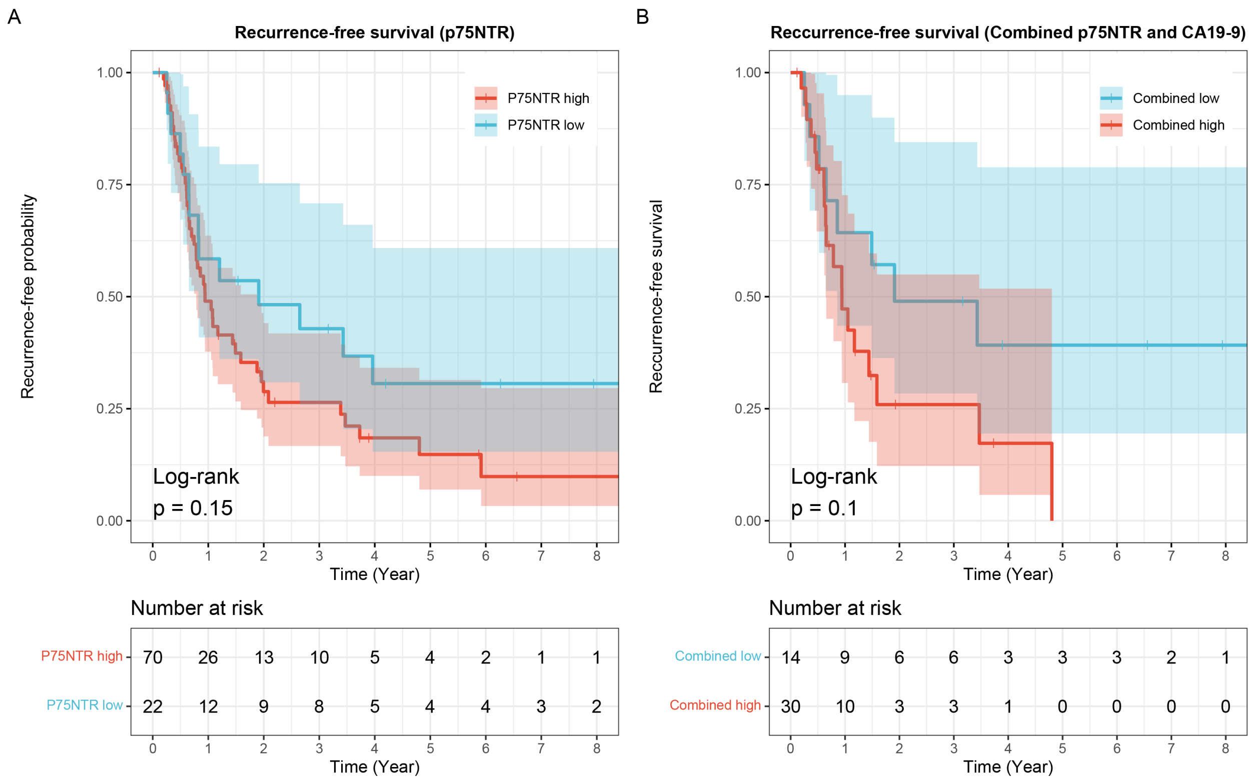

Fig. S14. Recurrence-free survival analysis of PDAC patients. (**A**) Kaplan-Meier curve analysis according to the p75NTR expression levels in the plasma-derived EVs from PDAC patients with and without NI. (**B**) Kaplan-Meier curve analysis of combination of plasma EV p75NTR expression (median cut-off separated into high and low group) and CA19-9 units per milliliter (U/mL) (low: <37 U/mL; high: >37 U/mL).

Supplementary tables

Table S1: The characteristics of PDAC patients for tissue- and plasma-derived EV evaluation in 3D migration assay

Clinical factors	Patients from UHD
**Total number, n**	53
**Sex**	
Male	19 (35.8%)
Female	33 (64.2%)
**Age** (years)	
Median (range)	68 (46-85)
**Cancer stage**	
I	7 (13.2%)
II	22 (41.5%)
III	13 (24.5%)
IV	11 (20.8%)
**Cancer grade**	
G1	0
G2	23 (43.4%)
G3	18 (34.0%)
Unknown	12 (22.6%)
**Resection margin**	
R0	37 (69.8%)
R1	4 (7.5%)
Unknown	12 (22.6%)
**Neoadjuvant chemotherapy**	
No	43 (81.1%)
Yes	10 (18.9%)
**Lymphatic invasion, L**	
No	27 (50.9%)
Yes	21 (39.6%)
**Venous invasion, V**	
No	33 (62.3%)
Yes	15 (28.3%)
Unknown	5 (9.4%)
**Neural invasion, Pn**	
No	10 (18.9%)
Yes	43 (81.1%)
**CA19-9**	
Median, U/mL(range)	112.0 (0.3-24839)
**Follow-up time**	
Median, days (range)	361.0 (4-3138)

Table S2: The characteristics of PDAC patients for overall survival and recurrence-free survival evaluation based on neural invasion status

Clinical factors	Patients from UHD
**Total number, n**	304
**Sex**	
Male	152 (50.0%)
Female	152 (50.0%)
Unknown	0
**Age** (years)	
Median (range)	69 (33-85)
Unknown	0
**Cancer stage**	
I	39 (12.8%)
II	192 (63.2%)
III	38 (12.5%)
IV	35 (11.5%)
Unknown	
**Cancer grade**	
G1	4 (1.3%)
G2	132 (43.4%)
G3	118 (38.8%)
Unknown	50 (16.4%)
**Resection margin**	
R0	236 (77.6%)
R1	48 (15.8%)
Unknown	18 (5.9%)
**Neoadjuvant chemotherapy**	
No	224 (77.6%)
Yes	60 (19.7%)
Unknown	20 (6.6%)
**Neural invasion, Pn**	
No	44 (14.5%)
Yes	260 (85.5%)
**CA19-9**	
Median, U/mL(range)	114.4 (0.3-38878)
Unknown (no. of patients)	30
**Follow-up time**	
Median, days (range)	462.0 (31-4543)

Table S3: The characteristics of PDAC patients for plasma-derived EV biomarker evaluation

Clinical factors	Patients from UHD
**Total number, n**	165
**Sex**	
Male	74 (44.8%)
Female	91 (55.2%)
Unknown	0
**Age** (years)	
Median (range)	68 (36-83)
Unknown	0
**Cancer stage**	
I	32 (19.4%)
II	82 (49.7%)
III	36 (21.8%)
IV	15 (9.1%)
**Cancer grade**	
G1	2 (1.2%)
G2	79 (47.9%)
G3	68 (41.2%)
Unknown	16 (9.7%)
**Resection margin**	
R0	106 (64.2%)
R1	17 (10.3%)
Unknown	42 (25.5%)
**Neoadjuvant chemotherapy**	
No	140 (84.8%)
Yes	22 (13.3%)
Unknown	3 (1.8%)
**Lymphatic invasion, L**	
No	53 (32.1%)
Yes	70 (42.4%)
Unknown	42 (25.5%)
**Venous invasion, V**	
No	84 (50.9%)
Yes	39 (23.6%)
Unknown	42 (25.5%)
**Neural invasion, Pn**	
No	25 (15.2%)
Yes	140 (84.8%)
**CA19-9**	
Median, U/mL(range)	120.6 (0.3-38878)
Unknown (no. of patients)	11
**Follow-up time**	
Median, days (range)	467.0 (34-4034)

Table S4: Primers for qRT-PCR

No	Gene name	Species	Forward primer (5' > 3')	Reverse primer (5' > 3')
**1**	*Gadph*	Murine	AGCTTGTCATCAACGGGAAG	CGGAGATGATGACCCTTTTG
**2**	*Npy*	Murine	CAAGAGATCCAGCCCTGAG	GTCTTCAAGCCTTGTTCTGG
**3**	*Ngfr*	Murine	CCTCATTCCTGTCTATTGCTC	CTGTTCCATCTCTTGAAAGCA
**4**	*Ngf*	Murine	AAGCTCACCTCAGTGTCTG	TACGCTATGCACCTCACTG
**5**	*Bdnf*	Murine	AATGGTGTCGTAAAGTTCCAC	GCAACCGAAGTATGAAATAACC
**6**	*Ntf5*	Murine	ACTACCTGTATCCTACAAAGGG	CTTGCACACCTGTCAACAG
**7**	*Gfra1*	Murine	CAAATTACATCTGCAGATCTCG	TAAGACAGTTGCTGACAGAC
**8**	*GAPDH*	Human	GACCCCTTCATTGACCTCAAC	TTGATTTTGGAGGGATCTCG
**9**	*NGFR*	Human	CCTCATCCCTGTCTATTGC	CTGTTCCACCTCTTGAAGG

494 **Table S5: Antibodies used for western blot**

No	Name	Company and catalog number	Dilution used
**1**	GAPDH	Cell Signaling Technology, #2118	1:1000
**2**	CD9	Abcam, ab92726	1:500
**3**	CD63	Abcam, ab68418	1:1000
**4**	CD81	Novus Biologicals, NBP2-20564	1:500
**5**	TSG101	Abcam, ab83	1:500
**6**	Alix	Cell Signaling Technology, #2171	1:500
**7**	Integrin β1	Cell Signaling Technology, #9699	1:500
**8**	EpCam	Abcam, ab71916	1:500
**9**	Fibronectin	Abcam, ab2413	1:2000
**10**	Ras^{G12D}	Cell Signaling Technology, #14429	1:1000
**11**	Calreticulin	Cell Signaling Technology, #2891S	1:1000
**12**	Syntenin	Abcam, ab133267	1:500
**13**	P75NTR	Cell Signaling Technology, #8238	1:500
**14**	NGF	Abcam, ab52918	1:1000
**15**	TrkA	Cell Signaling Technology, #2505S	1:500

495

Movies

Movie S1: Time-dependent uptake of Panc1-EVs by hSCs
